# ZrN-ZrO_*x*_N_*y*_ vs ZrO_2_-ZrO_*x*_N_*y*_ coatings deposited via unbalanced DC magnetron sputtering

**DOI:** 10.1038/s41598-021-98052-2

**Published:** 2021-09-23

**Authors:** Gloria I. Cubillos, Eduard Romero, Adriana Umaña-Perez

**Affiliations:** 1 Grupo de Materiales y Procesos Químicos, Departamento de Química, Universidad Nacional de Colombia, Bogotá, Colombia; 2 Grupo de Investigación en Hormonas, Departamento de Química, Universidad Nacional de Colombia, Bogotá, Colombia

**Keywords:** Engineering, Materials science

## Abstract

ZrN-ZrO${ }_{x}$N${ }_{y}$ and ZrO${ }_{2}$-ZrO${ }_{x}$N${ }_{y}$ coatings were deposited on 316L stainless steel substrates via the unbalanced DC magnetron sputtering technique in order to improve their corrosion resistance and evaluate their possible use as a coating biocompatible with bone cells. The composition, structure, morphology, and corrosion resistance were studied by sum means of x-ray photoelectron spectroscopy (XPS), x-Ray diffraction (XRD), scanning electron microscopy (SEM), and atomic force microscopy (AFM). The corrosion resistance was evaluated in 3.5 wt.% NaCl using potentiodynamic polarization (PL) and electrochemical impedance techniques (EIS). The ZrN-ZrO${ }_{x}$N${ }_{y}$ and ZrO${ }_{2}$-ZrO${ }_{x}$N${ }_{y}$ coatings exhibited barrier-type protection of the substrate against corrosion. The growth of mouse osteoblast cells was evaluated in the coating that exhibited the greatest resistance to corrosion, ZrO${ }_{2}$-ZrO${ }_{x}$N${ }_{y}$, finding that the cell viability was maintained, so this material can be considered to be a candidate for use in osteosynthesis processes.

## Introduction

Metallurgy is one of the research fields that directly influence industrial development worldwide. It underlies the chemical, automotive, oil, medical, construction, and food industries, among others^1–3^. For this reason, the scientific community is constantly looking for ways to produce new alloys with better mechanical, thermal, and corrosion-resistant properties, the latter being an area that is the subject of constant and growing research and development at an industrial level^3–7^. Depending on the field of application, the alloys must resist corrosion during their service time and simultaneously withstand environmental corrosion^2,5,7^. An example of this is the field of implants in dentistry or prosthetics in the osteosynthesis processes, where the metal not only must comply with the mechanical properties necessary to replace a bone piece or to promote the growth of bone cells but also must be harmless in a physiological environment in in vivo service^8,9^.

On the other hand, in production processes that involve heat exchangers with steam, this type of exchanger is a powerful source of corrosion, not only due to the oxygen dissolved in the water, which initiates the metal corrosion processes, but also due to the presence of CO${ }_{2}$ in solution, which contributes to the formation of carbonates. Additionally, the dissolved salts presents in the water causes pitting corrosion^10,11^.

Not only does the deterioration of a material occur due to the action of the environment on it or to interaction with the substances it must withstand when put into service, but additionally, the piece that material forms must withstand mechanical stress, with stress corrosion being the most serious, because it leads to the fracture of the piece^12,13^. Therefore, there is a need to develop alloys with protective additives, such as Cr, Mo, and Ni in the case of steels and Zn, Ni and Cu in that of aluminum, that improve the mechanical properties of the matrix and that, in contact with the atmosphere, produce protective oxides that act as a barrier to direct contact with the corrosive environment and isolate the metal from interaction with the atmosphere. Oxides are ceramic materials that are highly inert in acidic, alkaline, and neutral environments. However, these protective layers generated in situ, due to the conditions of use, are fragile and easily deteriorate, either through mechanical stress or through corrosion, exposing the metal matrix to degradation^3,5,14^.

Because of this, the development of the coatings and thin films industry goes hand in hand with that of the metallurgical industry. The coatings can be deposited with electrochemical techniques^15^, sol gel^16^, or high-vacuum techniques^17–20^, in which the deposition technique used allows improving the adherence of the coating and protects the matrix of the metal. The most often coatings and films used are ceramic^17,21–23^, metallic^24,25^ and metal-ceramic^26–28^, which are much more chemically inert than the oxides formed from the elements of the alloy. Cerium, lanthanum, titanium, zirconium, and chromium ceramics are deposited, in the form of either nitrides, oxides, or carbides^15,17,23^, so that the thin film is able to protect the substrate by isolating it from interaction with corrosive electrolytes.

The field of osteoimplantation is not exempt from the need for the combination of the mechanical properties of metal and the chemical inertness of ceramics. Today, many devices for long-term biomedical applications are constructed on a base of metal, but despite the wide variety of metallic materials available on the market, only a few fulfill the requirement of being biologically compatible with humans, e.g. titanium and its alloys, surgical-grade stainless steel (AISI 316L), and cobalt-chromium alloys^6,8^. However, in vivo the interaction of the metal in a physiological environment favors the solubilization of some of the alloyed metals, triggering the migration of ions such as Ni${ }^{2+}$ and Cr${ }^{3+}$ into the bloodstream^29^. To prevent the passage of these ions, as a defense mechanism, the body triggers irritation and subsequent inflammation, acidifying the environment and therefore considerably affecting the surface of the implant, favoring an increase in the concentration of toxic ions.

Although metallic biomaterials play a good biomechanical role, the presence of toxic ions causes an inflammatory response, releasing chemicals such as histamine, bradykinin, and prostaglandins, which activate macrophages that identify and attack these toxic substances. During the process, a series of enzymes are released that degrade certain mediators of inflammation, acidifying the physiological environment, which causes bone erosion and loss of material^30,31^. During the immune response, osteoclast formation is stimulated, increasing bone resorption and consequently producing areas of periprosthetic osteolysis, leading to aseptic loosening^32^. This chain of events forces the replacement of the implant and makes it necessary to use invasive corrective surgeries that affect the quality of life of the patient. Therefore, there is an urgent need to produce implants with greater durability and resistance to corrosion^30,33^.

The present research describes the synthesis of thin films of ZrN-ZrO${ }_{x}$N${ }_{y}$ and ZrO${ }_{2}$-ZrO${ }_{x}$N${ }_{y}$ coatings on stainless steel via unbalanced magnetron (UBM) sputtering in order to increase the corrosion resistance of stainless steel in highly corrosive environments. Due to the ceramic nature of the material, this nitride-oxynitride zirconium combination increases the corrosion resistance of the steel by one to two orders of magnitude. A structural characterization was performed using x-ray diffraction (XRD), and the morphological changes of the surface were determined through scanning electron microscopy (SEM) and atomic force microscopy (AFM). In addition, the corrosion resistance provided by the coating to two stainless steel samples of different composition was determined based on an analysis of their polarization resistance and their potentiodynamic polarization curves in a 3.5 wt.% NaCl solution. The coating with the best corrosion resistance was selected to evaluate its biocompatibility with the osteosynthesis process in vitro.

## Experimental procedure

### Substrate preparation and deposition conditions

AISI 316L and AISI 304 stainless steel specimens with areas of 2.0 $\times$ 2.0 cm were polished with 600 grit SiC. Prior to deposition, organic impurities were removed by washing and ultrasound with two solvents of different polarities, namely acetone and isopropanol. Non-commercial equipment was employed for growing the films via the UBM technique described previously^17^. The system contained a Gencoa sputter VT 100 unbalanced magnetron. The magnetic field configuration, KG = 1.00, was measured using a portable PHYWE teslameter with a Hall-effect probe. A discharge current of 170 mA and a discharge power of $\approx$ 340 W were applied, at a temperature of 387 K. The films were obtained from a 10 cm diameter and 0.6 cm thick Zr (99.9%) target (CERAC, Inc.). They were grown in an Ar atmosphere (99.99% purity) with a mixture 95% N${ }_{2}$/5% O${ }_{2}$ for the ZrN-ZrO${ }_{x}$N${ }_{y}$ coatings and 79% N${ }_{2}$/21% O${ }_{2}$ for the ZrO${ }_{2}$-ZrO${ }_{x}$N${ }_{y}$ coatings. The Ar and N${ }_{2}$ flow rates were set at 9.00 standard cubic centimeters/minute (sccm) and 3.00 sccm, respectively, and regulated using MKS mass flow independent controllers. The base pressure was less than 1.00 × 10^−3^ Pa. All the coatings were grown at 387 K, the sample target distance was set at 5 cm, and deposition time was 30 min, with a deposition rate of 10 nm/min. Under these experimental conditions, the coating’s thickness, determined by profilometry, was about 300 nm.

### Characterization of the coatings

The structural characterization of the films was performed via X-ray diffraction (XRD) with a Philips XPERT diffractometer operating at 30 kV and 20 mA, with Cu K$\alpha$ radiation ($\lambda$ = 0.1542 nm), using a step size of 0.05°. The surface morphology was characterized by imaging the secondary electrons using a Quanta 2000 scanning electron microscope operating at 15 kV and 10 mA.

The elemental composition and the chemical bonds of the thin films were measured via X-ray photoelectron spectroscopy (XPS) using a Kratos Analytical AXIS Ultra DLD system. The photoelectron spectra were excited by a soft X-ray Al K$\alpha$ (1486.6 eV) anode at a power of 120 W (10 mA, 12 kV). The specimens were sputtered with Ar${ }^{+}$ ions at 3 keV for 1 min in order to eliminate any surface contamination. The area of analysis of the samples was 700 × 300 microns. The C1s peak from the adventitious carbon-based contaminant with a value of 284.6 eV according to the literature was used as a reference to calibrate the XPS spectra in order to counteract the displacement in the spectra produced by using the charge neutralizer. The pressure in the chamber during the depth profile study was 1 × 10^−7^ Torr. The etching was done with the following parameters: 3 kV, 15 mA, and sample current 0.5 μA. The chemical composition of Zr, N, and O was determined on the basis of the area under the curve of the peaks of Zr3d${ }_{5/2}$ Zr3d${ }_{3/2}$, N1s, and O1s. The relative elemental quantities were calculated based on the area under the curve for each element’s XPS signal. Depth profile spectra were taken at 0, 1, 5, 10, 15, 20, 25 and 30 min. A 100 μm diameter analysis spot was used.

### Evaluation of the corrosion resistance

For the corrosion resistance study, 316L and 304 stainless steel was used as substrates for the ZrN-ZrO${ }_{x}$N${ }_{y}$ and ZrO${ }_{2}$-ZrO${ }_{x}$N${ }_{y}$ coatings, and a Solartron model 1287 potentiostat–galvanostat was used to measure the potentiodynamic polarization curves, in accordance with ASTM G5 (“Standard Reference Method for Making Anodic Potentiostatic and Potentiodynamic Polarization Measurements”). Prior to the corrosion tests, the samples were cleaned with isopropanol in ultrasound equipment. The electrochemical corrosion tests of the coating samples were performed in a three-electrode cell using a graphite electrode as a counter, an Ag/AgCl with 3 M KCl (+ 0.207 V vs. SHE) as reference electrode, and the coating samples as the working electrode. The study area of the coating samples was 1 cm^2^. The corrosion solution was 3.5 wt. % NaCl at room temperature. Potentiodynamic polarization tests were conducted by varying the potential between − 0.2 and 0.9 V at a rate of 0.1667 mV s^−1^. The data analyses were performed using Scribner software. CorrView was employed to study the LP curves. The corrosion tests were evaluated based on the polarization in the anodic region to determine the corrosion resistance of the film and to calculate the polarization resistance (${R}_{p}$) using the Simonds and Larson method^34,35^, in which ${R}_{p}$ is the slope of the curve obtained from the graphic voltage versus the density current.

In each test, 3 h of open circuit potential (${E}_{oc}$) was measured with the objective of letting the corrosion potential stabilize. Subsequently, EIS measurements were performed in potentiodynamic mode with a perturbation voltage of 20 mV vs. the corrosion potential.

The signal amplitude was 10 mV rms, and the measurement frequency ranged from 10^5^ to 10^−2^ Hz. Three repeated tests were performed for each set of measurements in order to ensure the reproducibility of the results.

### Biocompatibility study

Cell viability analyses were carried out in a 12-well, flat-bottom tissue culture containing the coatings on which osteoblast primary cells derived from the mouse cranial vault C57BL/6, available at the cell repository of the Hormone Research Group, were seeded at 1.2 × 10^4^ cells/well density in Dulbecco’s Modified Eagle Medium supplemented with 10% fetal bovine serum, ascorbic acid (50 μg mL^−1^), $\beta$-glycerophosphate (10 mM), and antibiotics (Thermo Fisher Scientist, USA). The cells were incubated at 37 °C in a 5% CO${ }_{2}$ humidified atmosphere for 72 h, allowing for cell adhesion and growth. Cell viability was determined using the MTT assay. For this, MTT solution was added to each well at 1.60 mg mL^−1^ final concentration, and the plates were incubated for 4 h at 37 °C. The formazan crystals were dissolved in 1.5 mL of 1% SDS in 0.01 M HCl, and the measurement of the absorbance (Abs) was performed at 570 nm in a Bio-Rad microplate reader. 100 μL of solution per well was used, and the percentage of cell viability was calculated according to the Eq. (1).

1 $$\mathrm{\%}\text{cell viability}=\frac{{\text{Abs}}_{570\hspace{0.25em}{\text{nm}}}\hspace{0.25em}{\text{sample}}-{\text{Abs}}_{570\hspace{0.25em}{\text{nm}}}\hspace{0.25em}{\text{blank}}}{{\text{Abs}}_{570\hspace{0.25em}{\text{nm}}}\hspace{0.25em}{\text{Control}}-{\text{Abs}}_{570\hspace{0.25em}{\text{nm}}}\hspace{0.25em}{\text{blank}}}\times 100$$

Additionally, osteoblasts were fixed on the surface of the material in accordance with Hosseini, S. et al.^32^. 7 × 10^3^ cells cm^2^ were seeded on the film, and the system was incubated at 37 °C and 5% CO${ }_{2}$ for 24 and 72 h with DMEM medium supplemented with 10% SFB. The samples were immersed in a 2.5% glutaraldehyde solution in 0.1 M PBS pH 7.4 for 4 h. Subsequently, they were washed in PBS in duplicate and dehydrated for 10 min by immersion in an aqueous ethanol solution of increasing concentrations: 30%, 50%, 60%, 80%, and 96%. The samples were immersed in 2 mL of 2.6-diamidine-2-phenylindole (DAPI) (SIGMA D9542) at a concentration of 1.00 mg mL^−1^, and after 12 h the samples were removed and washed three times with PBS. The DAPI-stained cells on coated and uncoated stainless steel were observed using confocal fluorescence microscopy (NIKON C1-Plus) and were captured in photographs. The morphology of the osteoblasts grown for 24 h attached to the film surface was determined with scanning electron microscopy (SEM) in a Quanta 2000® microscope operated at 15 kV and 10 mA. A sample of 600 grit AISI 316L steel polished with silicon carbide was used as the target. The samples were dried in a desiccator at room temperature for two hours before SEM microscopy analysis.

## Results

### Chemical composition of the coatings

Zirconium nitride-oxynitride coatings and zirconium oxide-oxynitride coatings were deposited on AISI 316L and AISI 304 stainless steel. The same deposition conditions used were: flow of gases, pressure, temperature and time deposit; the only variable was the composition of the reactive gases. For ZrN-ZrO${ }_{x}$N${ }_{y}$ coatings, a N${ }_{2}$/O${ }_{2}$ 95/05 mixture was employed as a reactive gas in order to favor the formation of nitride, since the formation of oxynitride is thermodynamically more stable^36^. For ZrO${ }_{2}$-ZrO${ }_{x}$N${ }_{y}$ coatings, N${ }_{2}$/O${ }_{2}$ 79/21 was employed as the reactive gas. For the surface chemical composition analysis, X-ray photoelectron spectra (XPS) were recorded. The chemical composition of the films deposited on each substrate (AISI 316L and AISI 304) did not exhibit significant differences. Shown here is the composition of the films deposited on AISI 316L. In order to acquire information about the in-depth distribution of the different species, a depth profile was shown on the layer deposited on the 316 L stainless steel substrate.

**Figure 1:**
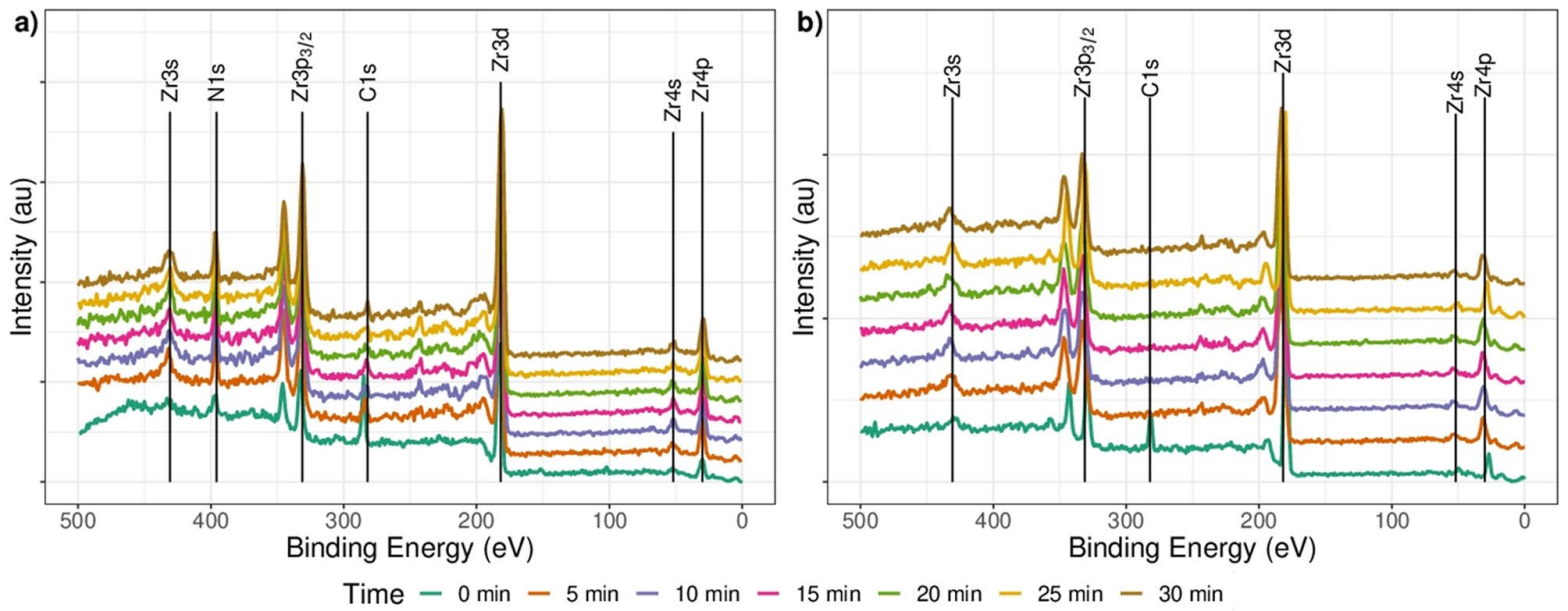
General spectrum for (**a**) ZrN-ZrO_*x*_N_*y*_ and (**b**) ZrO_2_-ZrO_*x*_N_*y*_ coating on AISI 316L. Depth profile 0, 5, 10, 15, 20, 25 and 30 min.

**Figure 2:**
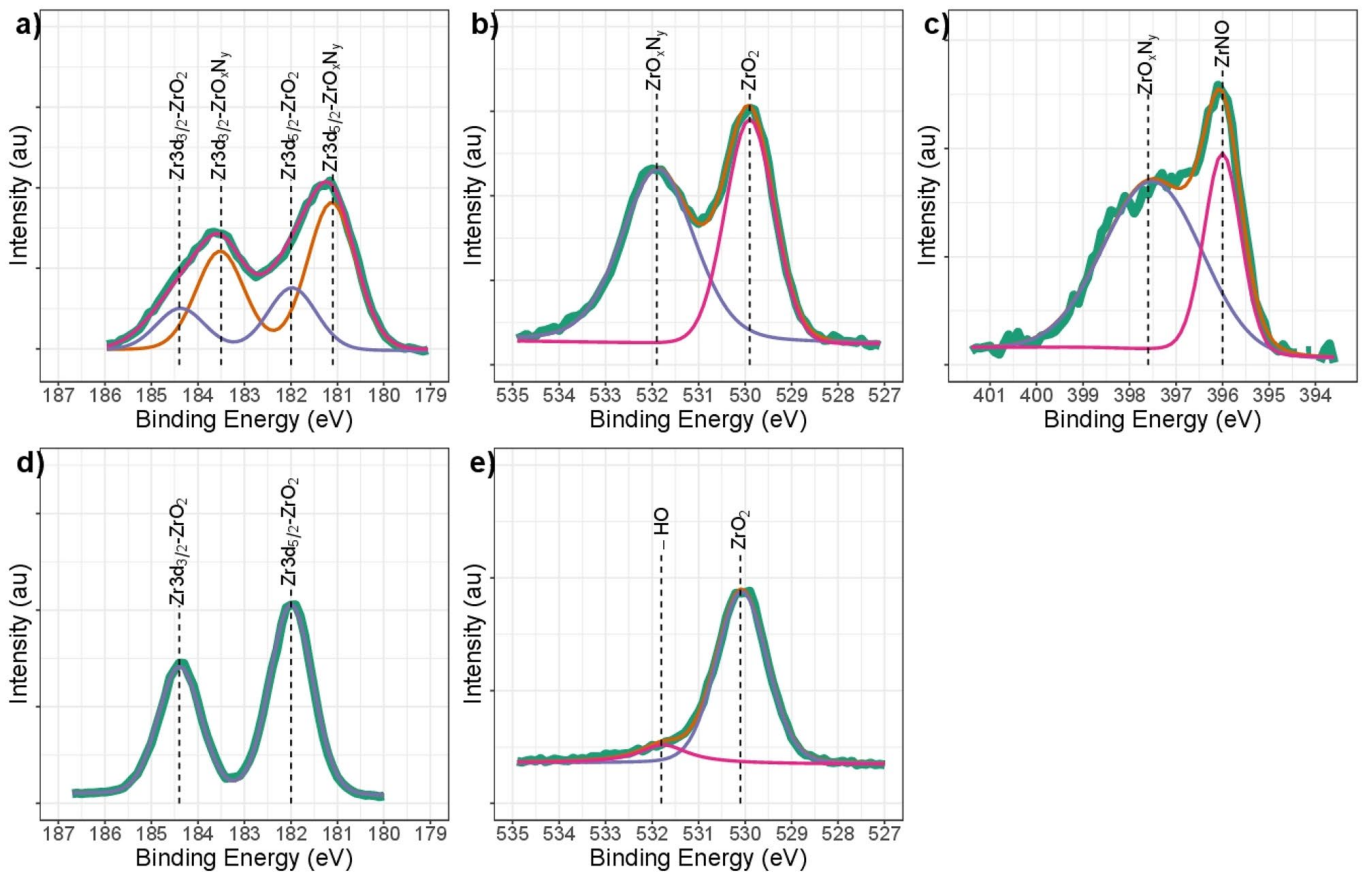
High-resolution XPS spectral analysis for the (**a**) Zr3d; (**b**) O1s and (**c**) N1s signals ZrN-ZrO_*x*_N_*y*_, (**d**) Zr3d and (**e**) O1S signals in ZrO_2_-ZrO_*x*_N_*y*_ coatings obtained by DC sputtering onto stainless steel. Spectrum recorded on surface after a 1 min sputter etching.

The XPS spectra were measured for samples of ZrN-ZrO${ }_{x}$N${ }_{y}$ and ZrO${ }_{2}$-ZrO${ }_{x}$N${ }_{y}$ at different sputtering times, 0, 1, 5, 10, 15, 20, 25, and 30 min, and the results are shown in Fig. 1(a- ZrN-ZrO${ }_{x}$N${ }_{y}$ and b- ZrO${ }_{2}$-ZrO${ }_{x}$N${ }_{y}$ coatings), where the signals for Zr, N, and C can be seen. With increasing depth (t = 0 to t = 30 min), the C1s signal decreases while the N1s signal increases. This is because the C1s signal is produced by the CO${ }_{2}$ in the atmosphere, while the N1s signal corresponds to the sample. Similar behavior was observed for the ZrO${ }_{2}$-ZrO${ }_{x}$N${ }_{y}$ coating, except that the signal on the surface of the N1s was undetectable. A more detailed analysis of the chemical composition based on the high-resolution XPS spectra is shown in Fig. 2.

Data corresponding to the Zr3d, O1s, and N1s spectral regions after 1 min of sputtering are shown in Fig. 2a–c for the ZrN-ZrO${ }_{x}$N${ }_{y}$ coating and Fig. 2d–e for the ZrO${ }_{2}$-ZrO${ }_{x}$ N${ }_{y}$ coating. In Fig. 2a, the spectrum for the film can be deconvoluted into two doublets, which show two different chemical environments for Zr3d. The binding energy for Zr3d${ }_{5/2}$ and Zr3d${ }_{3/2}$, centered at about 181.1 and 183.5 eV, respectively has been assigned by various authors to zirconium oxynitride. The binding energy for Zr3d${ }_{5/2}$ and Zr3d${ }_{3/2}$, centered at 182.0 and 184.4 eV, has been ascribed to ZrO${ }_{2}$, with a Zr3d${ }_{5/2}$-Zr3d${ }_{3/2}$ separation of 2.4 eV, in both cases within the experimental error^37–40^. Table 1 shows the values of the binding energies for the samples of ZrN-ZrO${ }_{x}$N${ }_{y}$ and ZrO${ }_{2}$-ZrO${ }_{x}$N${ }_{y}$.

**Table 1:** Summary of XPS binding energy values.

Sample	Compound	Binding energy			
		Zr3d_5/2_	Zr3d_3/2_	O1s	N1s
ZrN-ZrO_*x*_N_*y*_	ZrO_*x*_N_*y*_	181.1	183.5	531.9	397.7
	ZrO_2_	182.0	184.4	529.8	
	ZrNO				396.0
ZrO_2_-ZrO_*x*_N_*y*_	ZrO_2_	182.0	184.4	530.1	
ZrN-ZrO_*x*_N_*y*_-15 min*	ZrN	179.2	181.6		395.4
	ZrO_*x*_N_*y*_	181.0	183.4	530.7	396.5
	ZrO_2_	182.7	185.1	529.5	
ZrO_2_-ZrOZrO_*x*_N_*y-*_15 min*	ZrO_*x*_N_*y*_	180.0	182.4	531.4	398.3
	ZrOx	181.4	183.8		
	ZrO_2_	183.1	185.5	530.0	

*After 15 min sputter etching.

The presence of these phases is corroborated by the contribution at 529.8 and 531.9 eV in the O1s spectrum (Fig. 2b), which is characteristic of Zr-O bonds, the first associated with ZrO${ }_{2}$ and the second associated with ZrO${ }_{x}$N${ }_{y}$, where two types of oxygen can clearly be seen. O1s at B.E. 532 eV has also been reported for physisorbed water; however, after a 1 min sputtering, it was removed from the surface^40,41^. In addition, the N1s spectrum can also be deconvoluted into two peaks. The strongest one, at 397.7 eV, is assigned to ZrO${ }_{x}$N${ }_{y}$, and the weakest one, centered at 396.0 eV, to ZrNO^42,43^. This signal has been reported for Zr${ }_{2}$ON${ }_{2}$ by Q.N. Meng and collaborators and by Muneshwar, T and Cadien, K^41,43^. An analysis of the area under the curve for the binding energies of N1s indicated a chemical composition of 45% for the most nitrogen-substituted species of ZrNO and 54.3% for the most oxygenated species, ZrO${ }_{x}$N${ }_{y}$. The first one indicates stoichiometric ZrN${ }_{0.4}$O${ }_{0.7}$ and the second ZrN${ }_{0.2}$O${ }_{0.4}$. The two species of zirconium oxynitride exhibit very similar compositions, and therefore the binding energies for Zr3d do not allow distinguishing them, since in both cases the union is O-Zr-N; however, it is evident that the ZrN near the surface has been oxidized or oxynitrided by interaction with atmospheric oxygen^42^. By way of contrast, for the sample that was used in a reactive gas atmosphere, N${ }_{2}$/O${ }_{2}$ 79/21 for ZrO${ }_{2}$-ZrO${ }_{x}$N${ }_{y}$ coatings, the wide-scan XPS spectra only showed the presence of Zr and O signals for ZrO${ }_{2}$ on the surface (Fig. 2d and e, respectively). No N peaks were observed. Comparing the two types of coating, these results show that on the surface of the ZrN-ZrO${ }_{x}$N${ }_{y}$ coating, a nitride has oxidized to oxynitride and zirconia, while for the ZrO${ }_{2}$-ZrO${ }_{x}$N${ }_{y}$ coating, the oxynitride has oxidized to zirconia, this being the only phase present.

**Figure 3:**
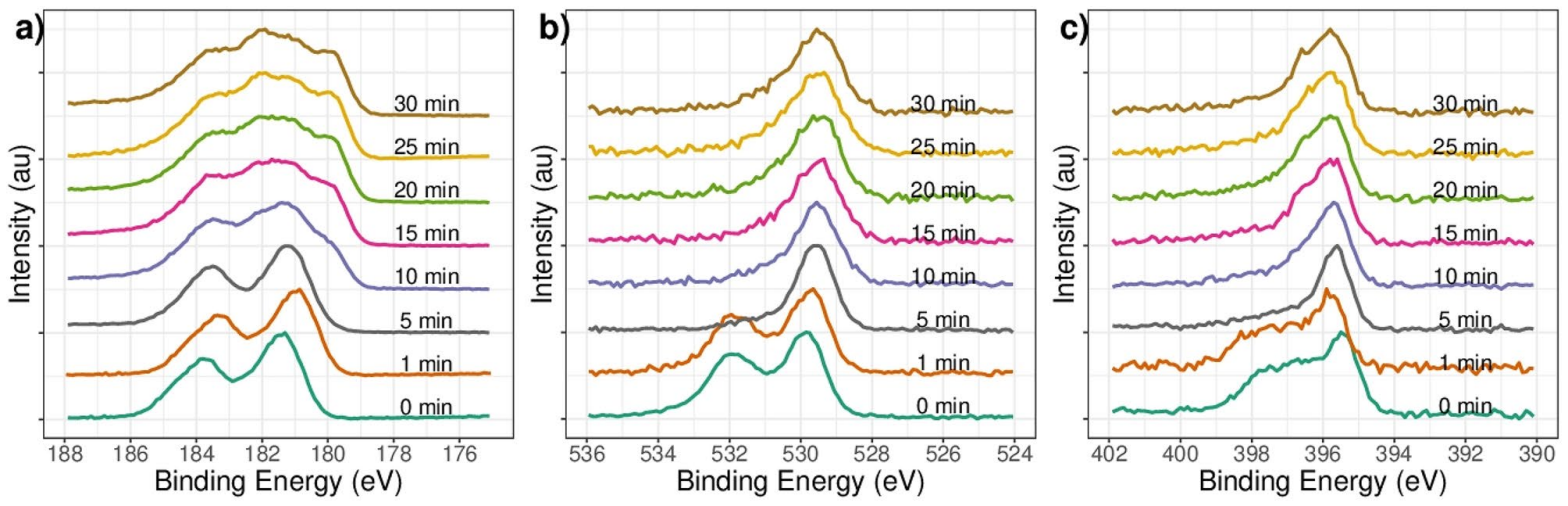
High-resolution XPS spectral analysis for the (**a**) Zr3d, (**b**) O1s and (**c**) N1s signals ZrN-ZrO_*x*_N_*y*_ coatings for different sputter etching times.

The depth profile for the ZrN-ZrO${ }_{x}$N${ }_{y}$ coating is shown in Fig. 3. The high-resolution XPS spectra for the zirconium 3d (Zr3d), oxygen 1 s (O1s), and nitrogen 1 s (N1s) for the films deposited onto 316L stainless steels are shown. Figure 3a) shows that after 1 min of cleaning, Zr3d is displaced toward lower binding energies. This displacement could be related to the increased nitrogen in the crystal’s structure. As the surface is stripped by sputtering with Ar${ }^{+}$ in five minutes periods, displacement of Zr3d signals towards lower binding energies can be observed, stabilizing the chemical composition after 10 min of sputtering approximately 50 nm from the surface. At this point, the binding energy at 178.5 eV could be related to the presence of ZrN. For O1s and N1s, the displacement towards lower binding energies as the surface is penetrated is of lower magnitude, and the contribution of the binding energy for O1s 532 eV (Fig. 3b) as the coating is penetrated decreases in intensity, while the signal at 395.5 for N1s increases in intensity. (Fig. 3c) In both cases, after 10 min of sputtering the chemical composition was constant. Similar results related to surface oxidation and change in concentration with bulk have been reported by N. Farkas and collaborators during ZrN oxidation^44^. For the ZrO${ }_{2}$-ZrO${ }_{x}$N${ }_{y}$ coating, the phases found in the depth profile were ZrO ${ }_{2}$, non-stoichiometric oxide ZrO${ }_{x}$, and ZrO${ }_{x}$N${ }_{y}$ (data not shown).

**Figure 4:**
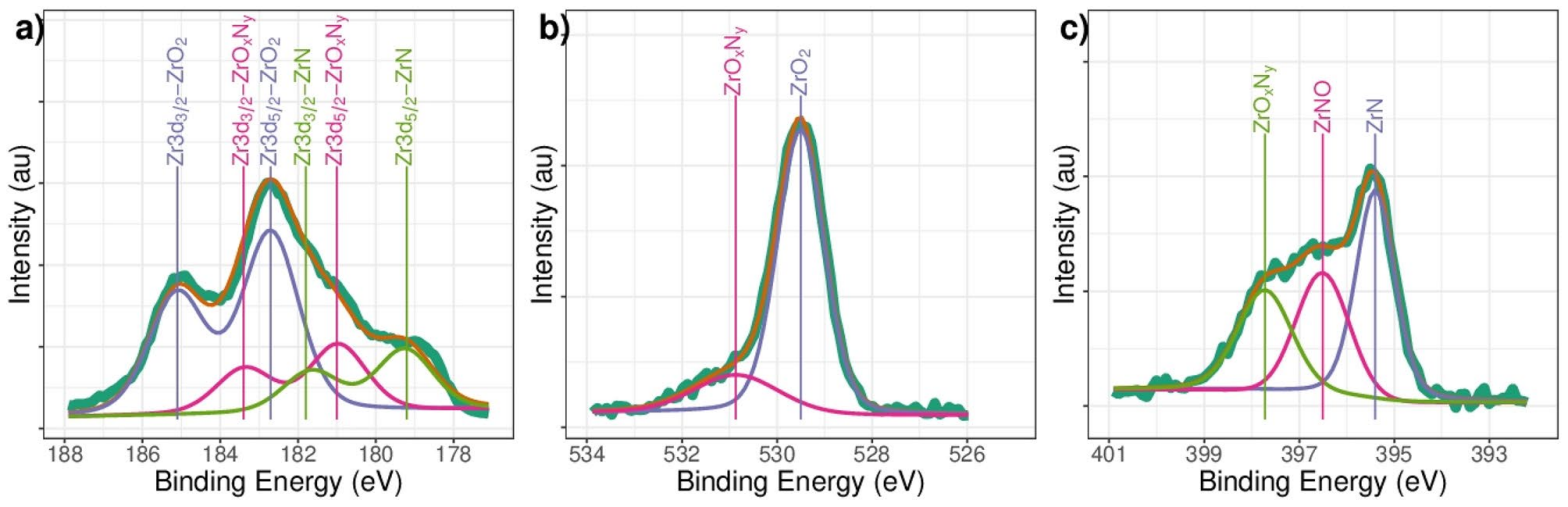
High-resolution XPS spectral analysis for the (**a**) Zr3d, (**b**) O1s and (**c**) N1s signals in ZrN-ZrO_*x*_N_*y*_ coatings after 15 min of etching.

The high-resolution spectra obtained after 15 min of sputtering with Ar${ }^{+}$ were characterized by a change in the chemical composition of the coating, as evidenced by a comparison of the high-resolution signals for Zr3d (Fig. 4a), O1s (Fig. 4b), and N1s (Fig. 4c). The binding energies associated with Zr 3d, O1s and N1s are presented in Table 1. A similar profile for Zr3d 182.8 eV signals was reported by Rizzo et al.^38^ in their synthesis of zirconium and titanium oxynitride films deposited on (100) Si wafers via the RF sputtering technique.

Associating these signals with the O1s and N1s, the binding energies at 529.5 and 530.8 eV correspond to zirconium oxide and zirconium oxynitride, respectively, with different compositions of nitrogen, the first associated with the species ZrNO corresponding to 41.2% and the second with ZrO${ }_{x}$N${ }_{y}$ with a contribution of 58.8%. For N1s, three binding energies can be seen, at 395.4 eV associated with ZrN, 396.5 eV with ZrNO, and 397.6 eV with ZrO${ }_{x}$N${ }_{y}$. The phases of the ZrNO and ZrO${ }_{x}$N${ }_{y}$ were identified as ZrO${ }_{0.4}$N${ }_{0.7}$ and ZrO${ }_{0.2}$N${ }_{0.4}$, respectively.

**Figure 5:**
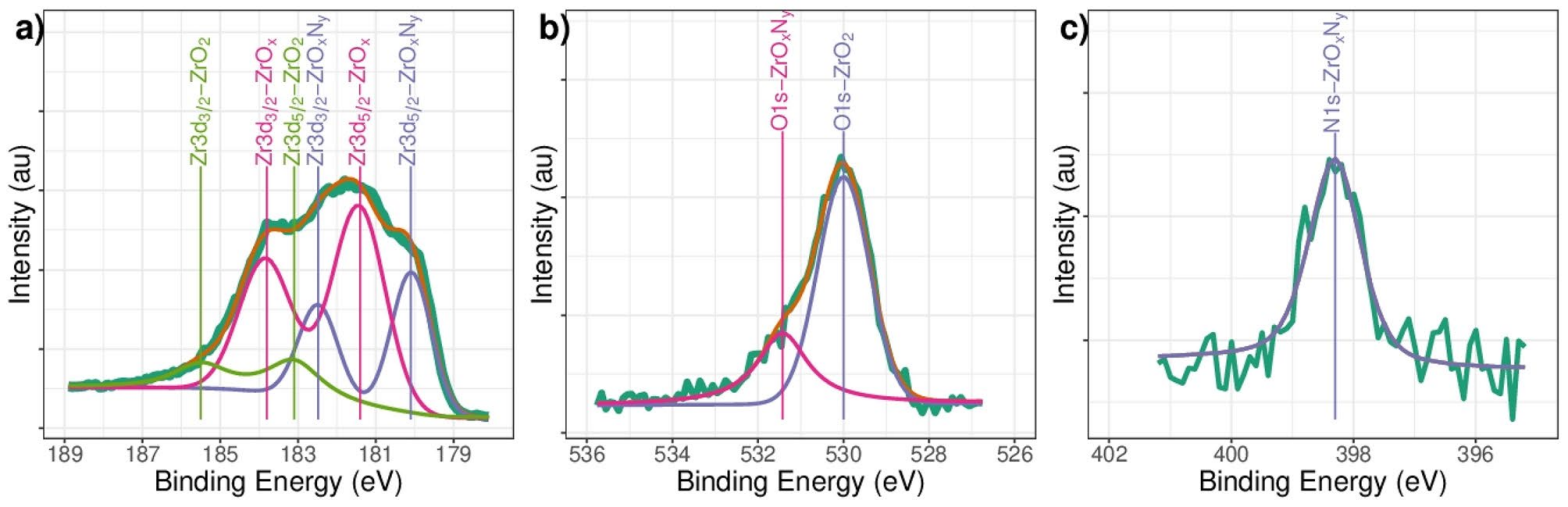
High-resolution XPS spectral analysis for the (**a**) Zr3d, (**b**) O1s and (**c**) N1s signals in ZrO_2_-ZrO_*x*_N_*y*_ coatings after 15 min of sputter etching.

Regarding the chemical composition of the ZrO${ }_{2}$-ZrO${ }_{x}$N${ }_{y}$ coatings after 15 min cleaning in Ar ${ }^{+}$, in Fig. 5a three phases can be seen: ZrO${ }_{x}$N${ }_{y}$, with Zr3d${ }_{5/2}$ binding energies at 180.0 eV and Zr3d${ }_{3/2}$ at 182.4, a new component, which can be attributed to a nonstoichiometric oxide ZrO ${ }_{x}$, with Zr3d${ }_{5/2}$ binding energies at 181.4 eV and Zr3d${ }_{3/2}$ at 183.8 eV, and ZrO${ }_{2}$, with binding energies for Zr3d${ }_{5/2}$ at 183.1 eV and Zr3d${ }_{3/2}$ at 185.5 eV. The presence of non-stoichiometric oxides is characteristic of coatings deposited via the sputtering technique, where the re-sputtering process can give rise to the formation of anion vacancies. The B.E. for these phases are confirmed by the B.E. for O1s at 530 eV, assigned to the Zr-O bond in ZrO${ }_{2}$ or Zr${ }_{x}$, and O1s at 531.3 eV, assigned to the O-Zr-N bond in ZrO${ }_{x}$N${ }_{y}$ (Fig. 5b). Unlike ZrN-ZrO${ }_{x}$N${ }_{y}$ coatings, a single nitrogen species is present at 398.3 eV, characteristic of ZrO${ }_{x}$N${ }_{y}$ (see Fig. 5c). In a more oxygen-rich atmosphere, the zirconia is the predominant phase, with 73.8%, while ZrO${ }_{x}$N${ }_{y}$ is at 26.2%.

The results obtained in this study indicate that the coating changes according to the chemical composition of oxygen and nitrogen in the reactive atmosphere. With a nitrogen content of 95%, ZrN-ZrO${ }_{x}$N${ }_{y}$ is deposited. If the film is deposited in an atmosphere of N${ }_{2}$/O${ }_{2}$ 79/21 on the surface, the only phase present is ZrO${ }_{2}$, and as it penetrates the coating, the composition is ZrO${ }_{2}$-ZrO${ }_{0.8}$N${ }_{0.2}$. As we have already mentioned, the composition of the film does not undergo major changes after 10 min of sputtering. From XPS, our research work related to thin films of zirconium oxynitrides has shown that atmospheric oxygen diffuses from the surface of the film to the bulk, favoring the formation of the most thermodynamically stable phase, the oxide. A detailed study was presented by Cubillos et al.^36^.

### Structure of the coatings

**Figure 6:**
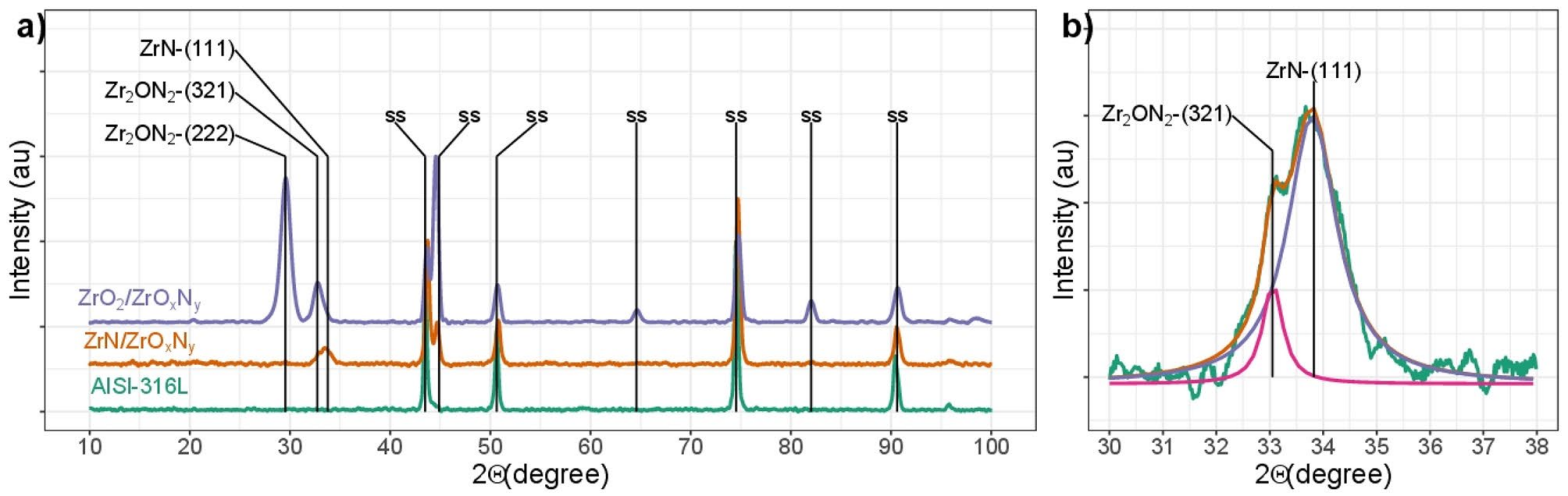
(**a**) XRD of ZrN-ZrO_*x*_N_*y*_, ZrO_2_-ZrO_*x*_N_*y*_ coatings on 316L stainless steels. Phases of AISI 316L is designated as (SS). (**b**) Peak decomposition between 30° and 40° for the ZrN-ZrO_*x*_N_*y*_ sample.

A change in the structure of the coatings can also be observed according to the composition of the reactive gas atmosphere. If the mixture of reactive gases used is N${ }_{2}$/O${ }_{2}$ 95/05, the crystal structure of the films is ZrN-ZrO${ }_{x}$N${ }_{y}$, shown as signals at 2$\theta$ 33.8 and 33.0 for ZrN and ZrO${ }_{x}$ N${ }_{y}$, respectively (see Fig. 6a in blue and deconvolution of the signal in Fig. 6b). The first signal was identified as the plane (111) in the cubic phase of ZrN according to the JCPDS 01–078-1420 standard, and the signal at 33.0 was identified as the plane 222 in the cubic phase of Zr${ }_{2}$ON${ }_{2}$ according to the JCPDS 00–050-1170 standard. The thin films show intense signals for (111) and (222) diffraction peaks, indicating a more strongly preferred (111) orientation in ZrN and (222) in ZrO${ }_{x}$N${ }_{y}$.

For N${ }_{2}$/O${ }_{2}$ 79/21, the crystal structure of the films is ZrO${ }_{x}$N${ }_{y}$, shown as signals at 2$\theta$ 29.9 and 33.0 according to JCPDS 00–050-1170 (Fig. 6a). However, the chemical composition of this coating showed the presence of ZrO${ }_{2}$, and taking into account that cubic zirconia also exhibits the signal for the plane (111) at 2$\theta$=30.1 (JCPDS 00–049-1642), both phases may be present.

### Surface morphology

**Figure 7:**
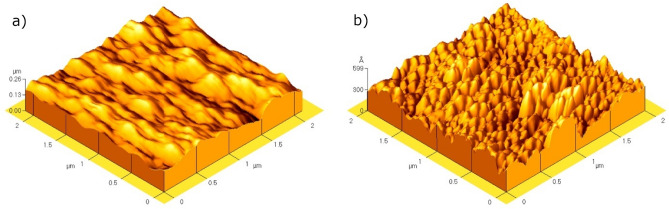
AFM micrograph image of the ZrO_*x*_N_*y*_ film. (**a**) ZrO_2_-ZrO_*x*_N_*y*_, (**b**) ZrN-ZrO_*x*_N_*y*_ deposited on 316L stainless steel. (**a**) XRD of ZrN-ZrO_*x*_N_*y*_, ZrO_2_-ZrO_*x*_N_*y*_ coatings on 316L stainless steels.

Figure 7 shows the surface morphology on the basis of AFM. For both types of coatings, the film is characterized as homogeneous. However, a larger formation of grains with distinguishable edges can be observed in Fig. 7a for ZrN-ZrO${ }_{x}$N${ }_{y}$, which suggest a denser nucleation process compared to ZrO${ }_{2}$-ZrO${ }_{x}$N${ }_{y}$ Fig. 7b), which is characterized by its grains of larger volume with a denser structure, where the growth of the film it does not allow differentiating the limits between them. The grain size of ZrO${ }_{2}$-ZrO${ }_{x}$N${ }_{y}$ is 1.8 times greater than that of the ZrN-ZrO${ }_{x}$N${ }_{y}$ film (Table 2). The difference in the growth of the two coatings may be due to their different factors: the orientation of the substrate with respect to the target and differences in the morphology of the substrate surface, which although polished to the same particle size can be heterogeneous. This is supported by the average roughness values shown in Table 2 and the parameters associated with the formation of grains and number of grains/μm^2^. The roughness of ZrO${ }_{2}$-ZrO${ }_{x}$N${ }_{y}$ film is 6.4 times greater than that of the ZrN-ZrO ${ }_{x}$N${ }_{y}$ film (Table 2).

**Table 2:** Parameters characteristic the morphology of the films.

Sample	Roughness		Grain size		# grains per area	
	(nm)	sd (nm)	(nm)	sd (nm)	(μm^2^)	sd (μm^2^)
ZrO_2_-ZrO_*x*_N_*y*_	9.6	0.17	381	46	9	2
ZrN-ZrO_*x*_N_*y*_	1.5	0.14	216	17	27	4

### Corrosion resistance

#### Polarization curves

**Figure 8:**
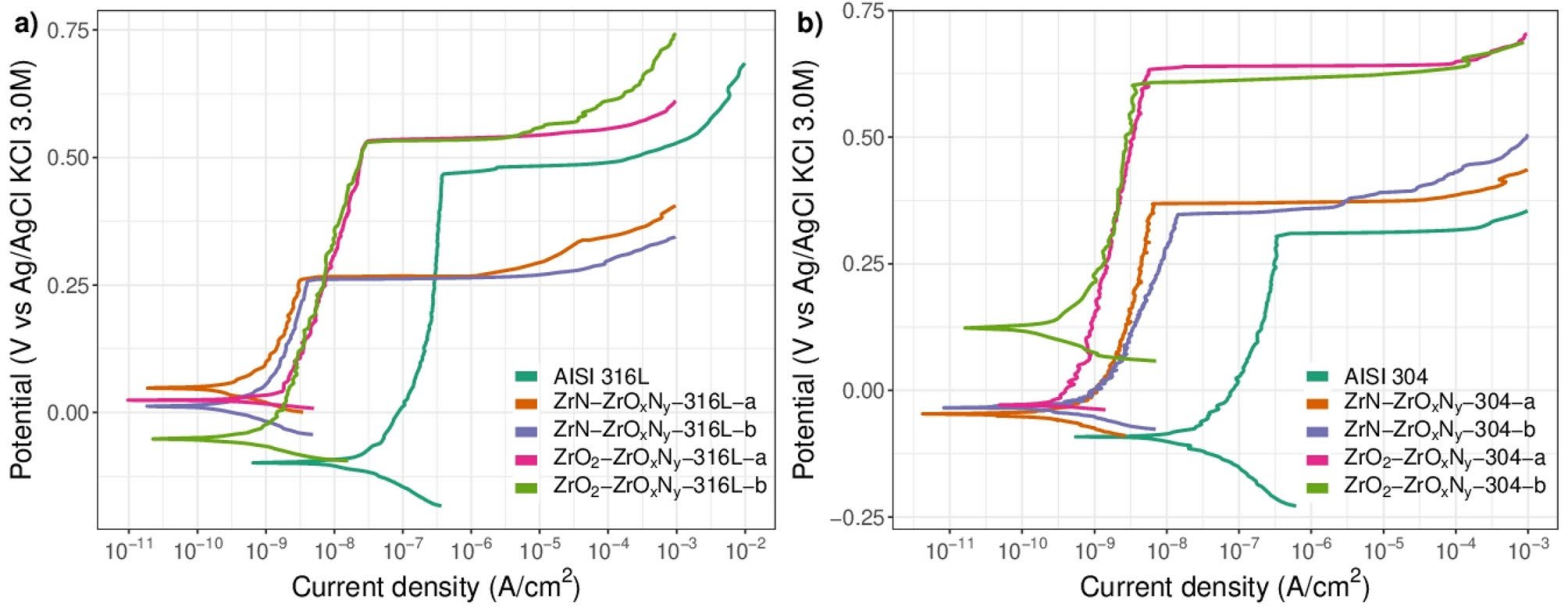
Linear polarization curves of the ZrO_2_-ZrO_*x*_N_*y*_, and ZrN-ZrO_*x*_N_*y*_ deposited on (**a**) AISI 316 L and (**b**) AISI 304.

For this investigation, of the degree of protection offered by the coating on steel, deposition of the film on two stainless steels with different compositions was evaluated: AISI 316L and AISI 304. The ability of the coatings to protect the steel against corrosion was evaluated using the potentiodynamic polarization curves and the ${R}_{p}$ values obtained for them. The results are shown in Fig. 8a and b and Table 3. The magnitude of the ${R}_{p}$ is indicative of the degree of protection provided by the coating on the steel and represents a reduction in the electrochemical activity of the system.

**Table 3:** Values average of the parameters of the corrosion test.

Sample	${j}_{corr}$	${E}_{corr}$	${R}_{p}$	CR	PNP	PNP-CR
	(nA cm^−2^)	(mV)	(MΩ cm^2^)	(mmpy)/ × 10^−6^	(V)	(V)
AISI 304	33.9	− 101	0.778	260	0.304	0.405
ZrO_2_-ZrO_*x*_N_*y*_-304	1.83	− 82.0	40.1	7.14	0.620	0.702
ZrN-ZrO_*x*_N_*y*_-304	0.932	− 45.6	29.1	7.24	0.357	0.402
AISI 316L	41.3	− 109	0.630	321	0.464	0.573
ZrO_2_-ZrO_*x*_N_*y*_-316L	2.82	− 43.1	12.4	22.0	0.531	0.574
ZrN-ZrO_*x*_N_*y*_-316L	0.6.98	− 20.2	37.8	5.69	0.260	0.280

${j}_{corr}$ = corrosion current density; ${E}_{corr}$ = corrosion potential; ${R}_{p}$ = polarization resistance; CR = corrosion rate; mmpy = millimeters per year; PNP = pitting nucleation potential.

For steel coated with both ZrN-ZrO${ }_{x}$N${ }_{y}$ and ZrO${ }_{2}$-ZrO${ }_{x}$N${ }_{y}$, it can be seen that the film of ceramic material increases the resistance to corrosion of the steel by one or two orders of magnitude, as can be seen in Table 3 from the corrosion current density (${j}_{corr}$), the resistance to polarization (${R}_{p}$), and the corrosion rate (CR). On AISI, the ZrO${ }_{2}$-ZrO${ }_{x}$N${ }_{y}$-316L coating decreases its corrosion current density and corrosion rate by one order of magnitude relative to bare steel and increases polarization resistance by two orders of magnitude. The average value of the corrosion parameters obtained for each duplicate is shown in Table 3 in order to facilitate analysis of the results. On AISI 304, ZrO${ }_{2}$-ZrO${ }_{x}$N${ }_{y}$ coatings exhibit similar behavior, the difference being that the corrosion current density is lower than that of steel by an order of magnitude. For ZrN-ZrO${ }_{x}$N${ }_{y}$ coatings, the protection afforded by the coating on the two types of steel is very similar and superior by an order of magnitude to that of ZrO${ }_{2}$-ZrO${ }_{x}$N${ }_{y}$ coatings, decreasing the current density and the corrosion rate by two orders of magnitude relative to bare steel and increasing ${R}_{p}$ by two orders of magnitude compared to bare steel.

**Figure 9:**
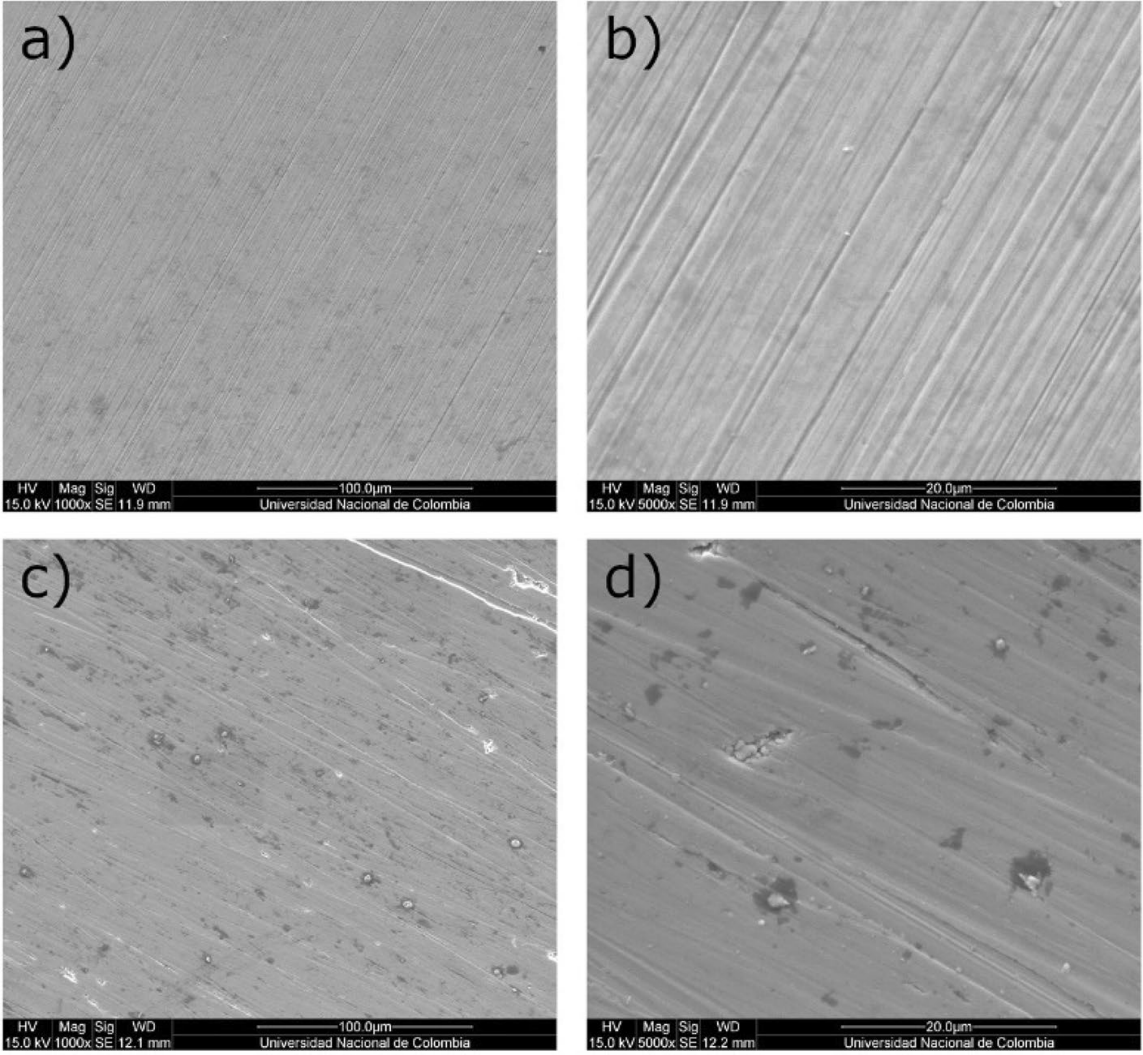
SEM micrograph showing the morphology of the ZrN-ZrO_*x*_N_*y*_-316L coatings (**a**, **b**) before corrosion; (**c**, **d**) after corrosion in 3.5 wt. % NaCl solution.

**Figure 10:**
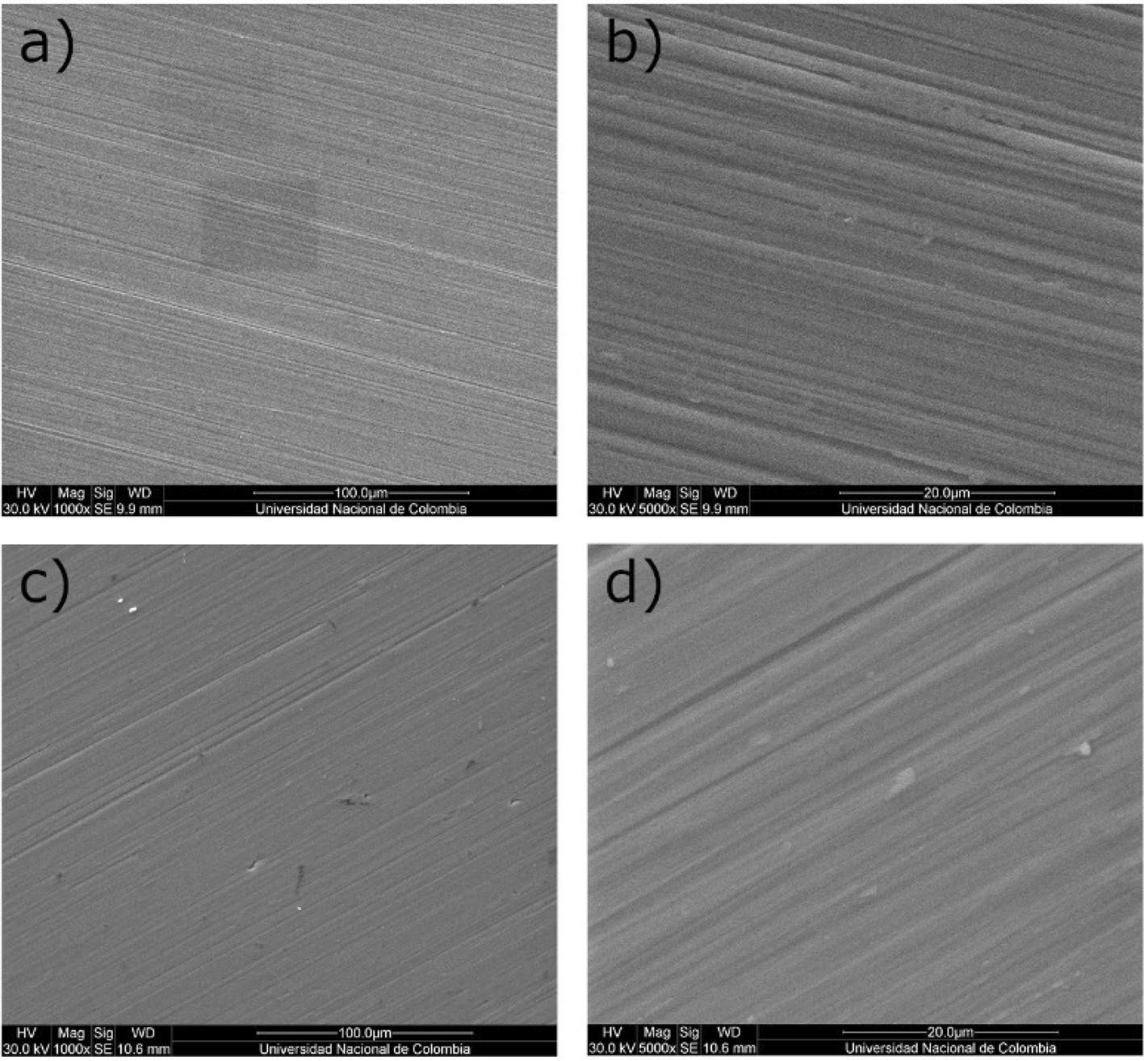
SEM micrograph showing the morphology of the ZrO_2_-ZrO_*x*_N_*y*_-316L coatings (**a**, **b**) before corrosion; (**c**, **d**) after corrosion in 3.5 wt. % NaCl solution.

The results of the polarization curves show that the resistance to polarization against the electrolyte is greater with a film of ZrN-ZrO${ }_{x}$N${ }_{y}$. However, the potential for pit nucleation and therefore the passivation zone are diminished in the ZrN-ZrO${ }_{x}$N${ }_{y}$-stainless steel system with respect to ZrO${ }_{2}$-ZrO${ }_{x}$N${ }_{y}$-stainless steel. For the ZrN-ZrO${ }_{x}$N${ }_{y}$ coating deposited on AISI 316 L, the pitting nucleation potential (PNP) decreases by 0.2 V, and so does the passivation zone (${E}_{corr}-PNP$) compared with the PNP of the substrate, while for the ZrO${ }_{2}$-ZrO${ }_{x}$N${ }_{y}$ coating it increases by approximately 0.067 V in comparison with the substrate. On AISI 304, the PNP and the passivation zone are of the same order of magnitude as for ZrN-ZrO${ }_{x}$N${ }_{y}$ coatings, while for ZrO${ }_{2}$-ZrO${ }_{x}$N${ }_{y}$ it increases by 0.3 V compared to steel. These results are reflected in the morphology of the specimens observed through SEM before and after the polarization test in Figs. 9 and 10.

The SEM analysis of the two systems before and after the corrosion test in a 3.5 wt.% NaCl solution is shown in Figs. 9 and 10, comparing the images for the two systems presented, ZrN-ZrO${ }_{x}$N${ }_{y}$-316L and ZrO${ }_{2}$-ZrO${ }_{x}$N${ }_{y}$-316L, at two different magnifications. Micropores and microcracks can be observed in the surface morphology of the ZrN-ZrO${ }_{x}$N${ }_{y}$ film deposited on 316L stainless steel (Fig. 9a, b), which would explain the decrease in the pitting nucleation potential in the PL and the fact that after the corrosion test, the damage generated on the surface of the coating is greater (Fig. 9c, d), with zones of film delamination. Before the corrosion test, the ZrO${ }_{2}$-ZrO${ }_{x}$N${ }_{y}$ coating exhibited very few imperfections on its surface, Fig. 10a, b, and the damage caused after the corrosion is less severe compared with the ZrN-ZrO${ }_{x}$N${ }_{y}$, Fig. 10c, d.

Similar results can be observed on the surface of the coating deposited on AISI 304 following the corrosion test. While it is true that the ZrN-ZrO${ }_{x}$N${ }_{y}$ coating was deposited in a nitrogen-rich N${ }_{2}$/O${ }_{2}$ 95/05 atmosphere, the chemical composition determined via XPS shows that it is formed by a mixture of ZrN, ZrO${ }_{x}$N${ }_{y}$, and ZrO${ }_{2}$ phases generated by the interaction with atmospheric oxygen, according to the chemical reactions presented in Eqs. (2) and (3).

2 $$Zr{N}_{y}\left(s\right)+\left(x/2\right){O}_{2}\left(g\right)->Zr{O}_{x}{N}_{y}\left(s\right)$$

3 $$Zr{O}_{x}{N}_{y}\left(s\right)+{O}_{2}\left(g\right)->Zr{O}_{2}\left(s\right)+{N}_{y}{O}_{x}\left(g\right)$$

**Figure 11:**
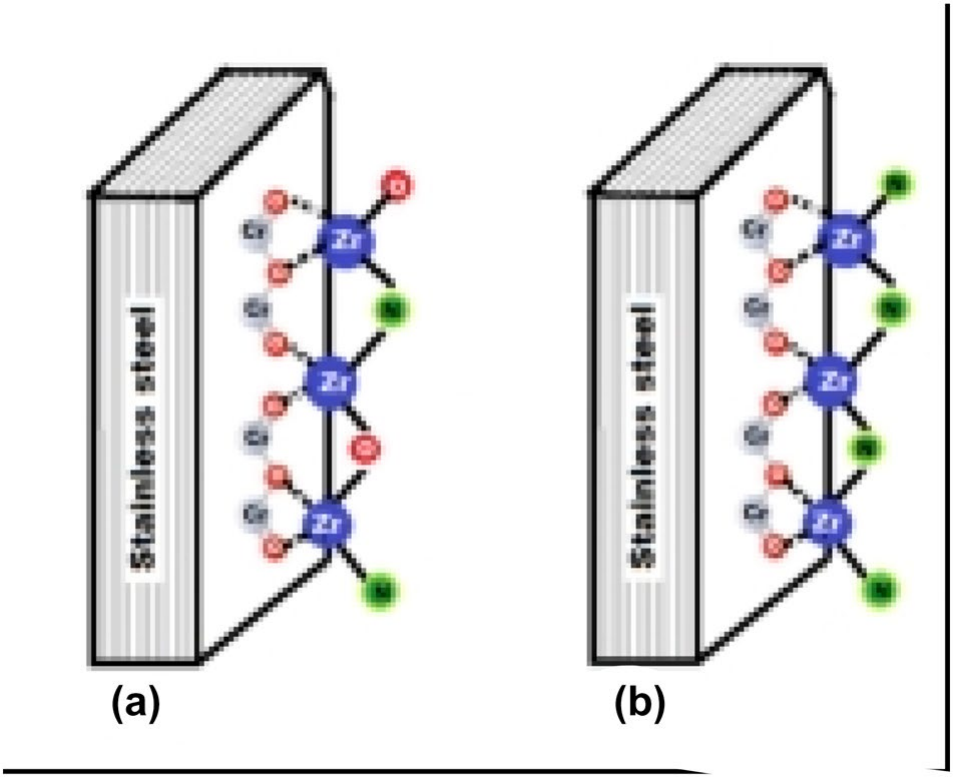
Schematic representation of the substrate-coating interaction forces (**a**) ZrO_2_-ZrO_*x*_N_*y*_ (**b**) ZrN-ZrO_*x*_N_*y*_ coatings.

Oxidation of nitride to zirconia increases unit cell volume by 39.9% (${V}_{cell}$ZrN = 96.39 Å^3^, ${V}_{cell}$ ZrO${ }_{2}$ = 134.85 Å^3^) (JCPDS 00-049-1642 and 01-078-1420). On the other hand, the film deposited using the sputtering technique grows de novo from its chemical elements, where initially the vaporized zirconium is deposited on the substrate. At this point, both the adhesion and the substrate-coating’s crystalline structure depend on the interaction forces of the zirconium with the alloying elements of the substrate. In Fig. 11a and b, as an example, the interaction forces of Zr with Cr${ }_{2}$O${ }_{3}$ of the passivating layer present on the surface of the steel are outlined.

**Figure 12:**
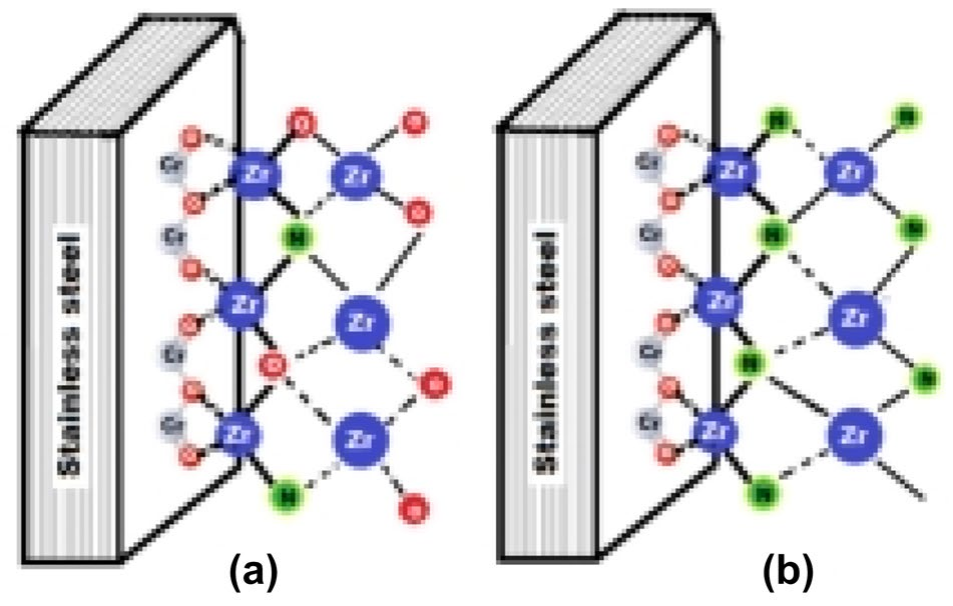
Schematic representation of the substrate-coating interaction forces. (**a**) ZrO_2_-ZrO_*x*_N_*y*_ (**b**) ZrN-ZrO_*x*_N_*y*_ coatings.

Once the Zr adheres to the substrate, it reacts in the solid phase with the reactive gases N${ }_{2}$ and O${ }_{2}$. In an atmosphere enriched in oxygen, N${ }_{2}$/O${ }_{2}$ 79/21, the predominant Van der Waals forces would be Zr-O, since the predominant anion according to the XPS and XRD results is oxygen. If the atmosphere were enriched in nitrogen, N${ }_{2}$/O${ }_{2}$ 95/05, the interaction forces would be predominantly Zr-N, weaker than Zr-O according to the higher electronegativity of oxygen (O ${ }_{2}$= 3.44, N${ }_{2}$= 3.04). During the growth of the film, the Zr-anion ratio is the same for the two types of coating, Fig. 12a and b. As mentioned before, the formation of ZrO${ }_{x}$N${ }_{y}$ in the film ZrN-ZrO${ }_{x}$N${ }_{y}$ occurs by diffusion of atmospheric oxygen from the surface to the substrate, which implies that the predominant Van der Waals forces in the substrate-coating interface would be Zr-N, and this would be related to the greater delamination of the ZrN-ZrO${ }_{x}$N${ }_{y}$ coating during the corrosion test, versus ZrO${ }_{2}$-ZrO${ }_{x}$N${ }_{y}$, as was observed through SEM.

#### Electrochemical impedance spectroscopy

**Figure 13:**
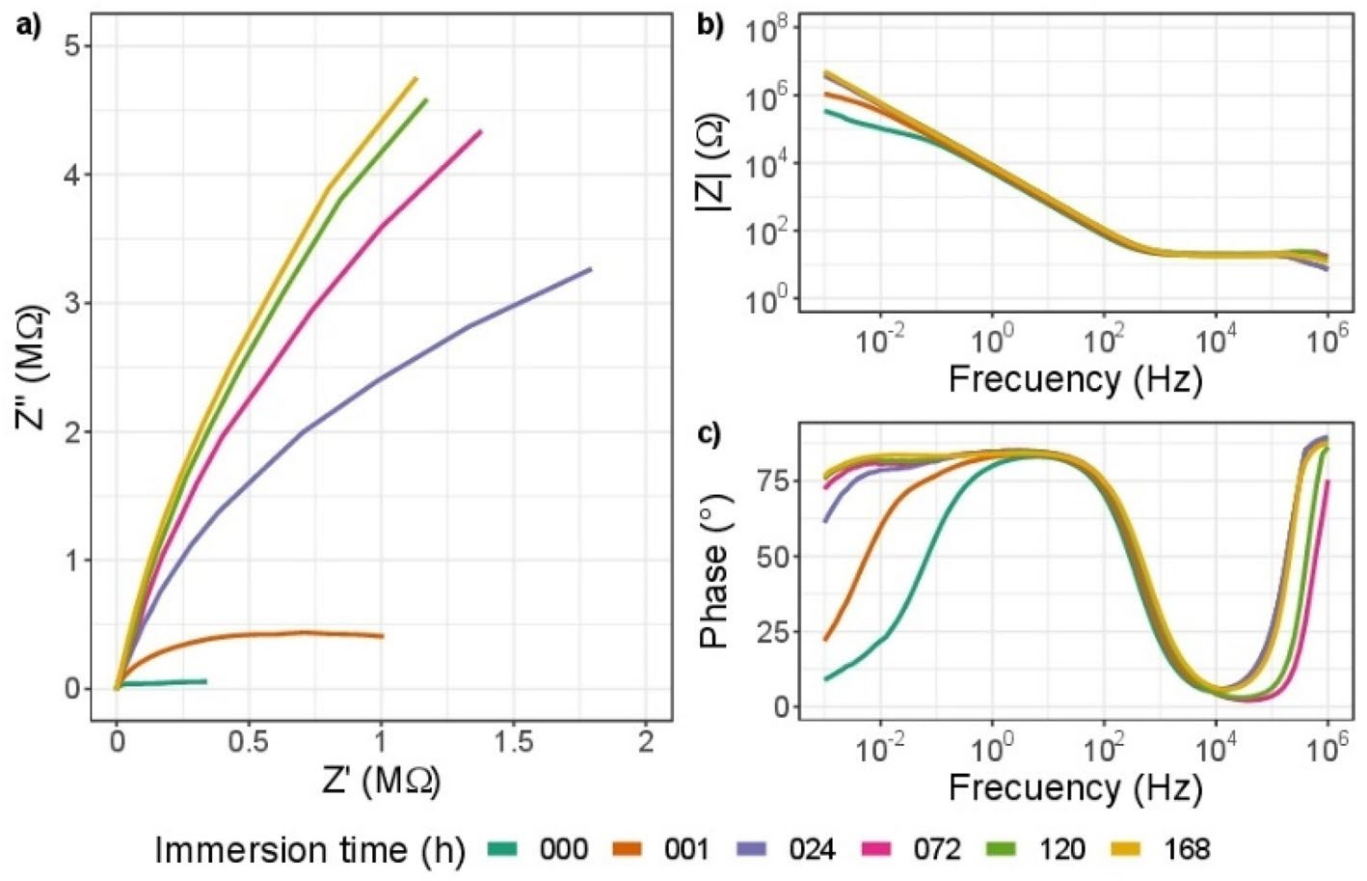
EIS diagram for impedance modulus obtained after different immersion times in 3.5% wt NaCl solution for stainless steel 316L (**a**) Nyquist impedance (**b**) Bode impedance and (**c**) Bode phase angle plots.

**Figure 14:**
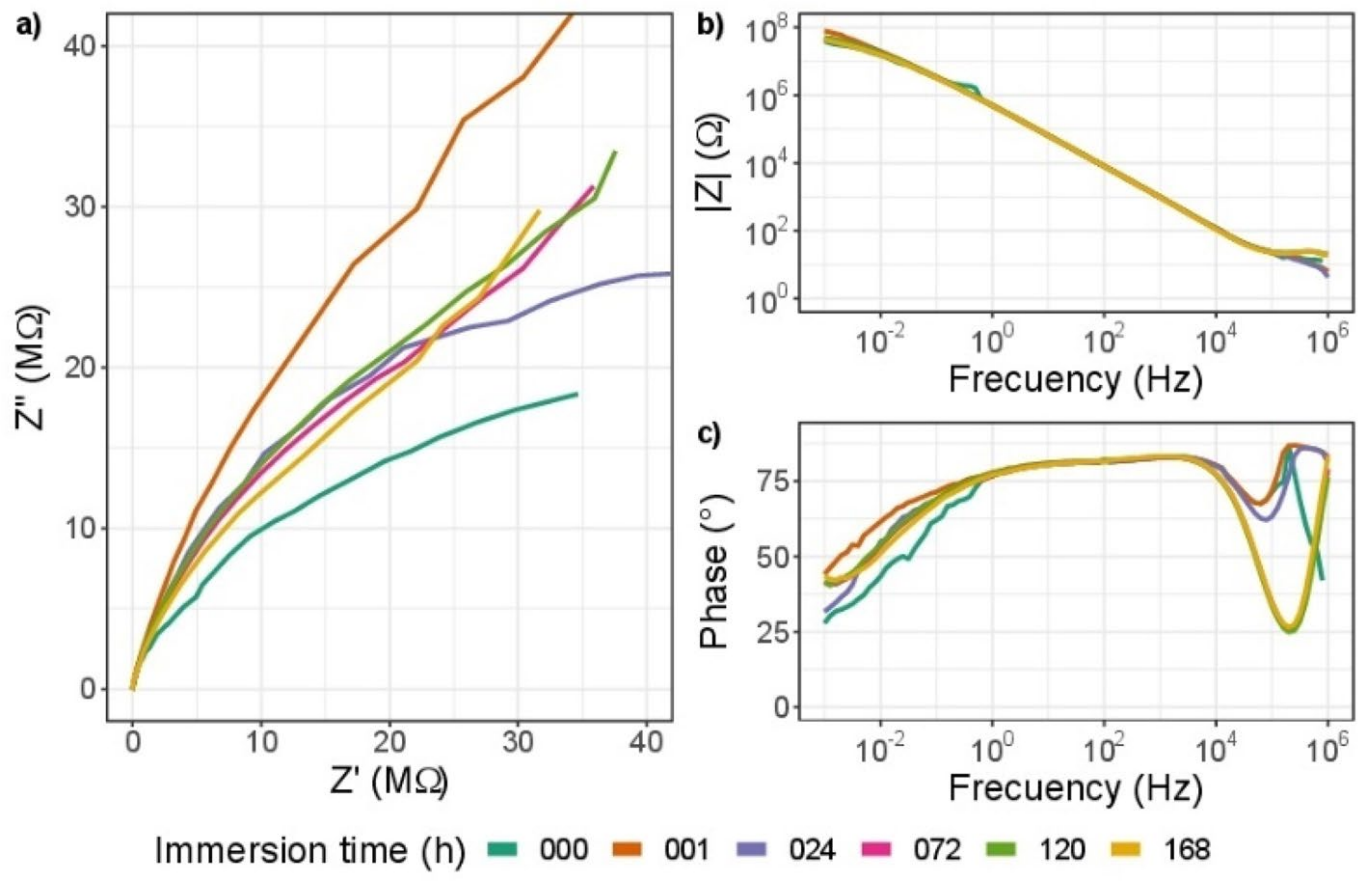
EIS diagram for impedance modulus obtained after different immersion times in 3.5% wt NaCl solution for ZrO_2_-ZrO_*x*_N_*y*_, (**a**) Nyquist impedance (**b**) Bode impedance and (**c**) Bode phase angle plots.

**Figure 15:**
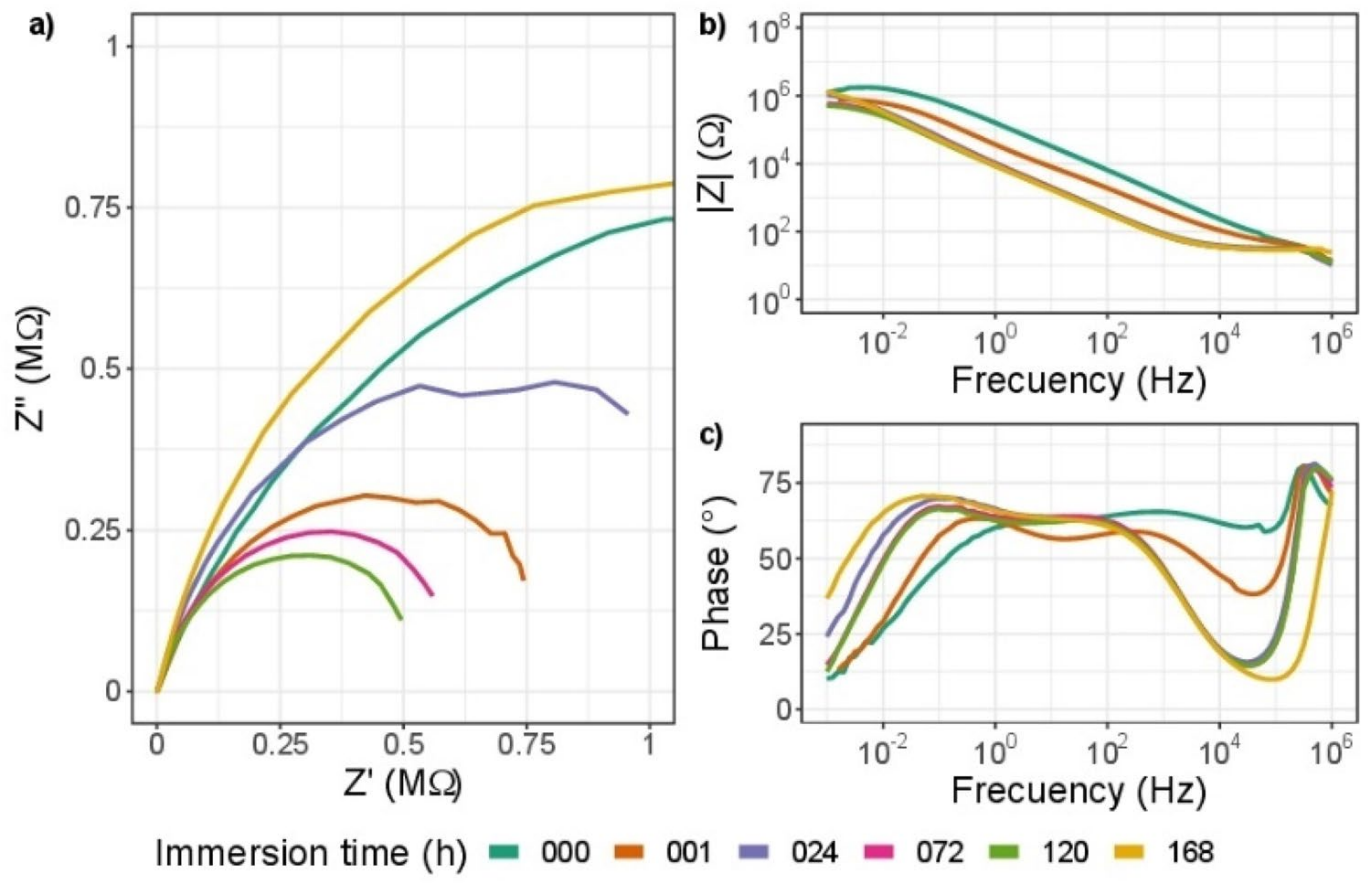
EIS diagram for impedance modulus obtained after different immersion times in 3.5% wt NaCl solution for ZrN-ZrO_*x*_N_*y*_, (**a**) Nyquist impedance (**b**) Bode impedance and (**c**) Bode phase angle plots.

**Figure 16:**
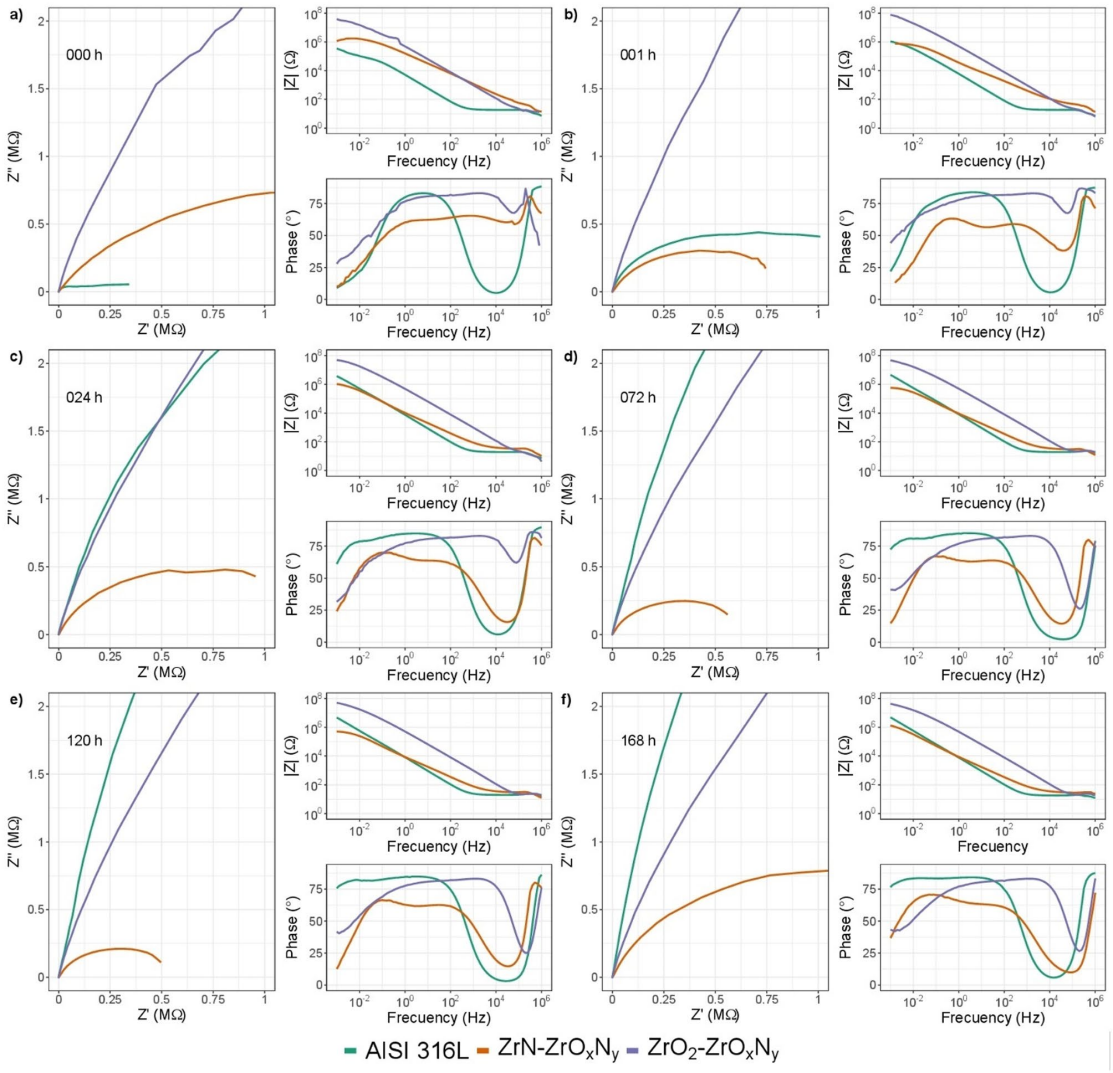
Comparative diagram for impedance modulus obtained after different immersion times in 3.5 wt. % NaCl solution for AISI 316L; for ZrN-ZrO_*x*_N_*y*_ and for ZrO_2_-ZrO_*x*_N_*y*_ coatings.

The efficiency of a coating to be used in an osteo implant is directly related to its chemical inertness and biocompatibility. The electrochemical impedance spectroscopy (EIS) study of surgical-grade AISI 316L bare steel coated with ZrN-ZrO${ }_{x}$N${ }_{y}$ or ZrO${ }_{2}$-ZrO${ }_{x}$N${ }_{y}$ provides information related to the corrosion behavior of the steel-coating system that is put into service. The results obtained after exposure to the corrosive action of a 3.5 wt. % NaCl solution for different immersion times are shown here: 1, 24, 72, 120, and 168 h, in Figs. 13, 14, 15 and 16. The same color reference is used for the same exposure time, in order to facilitate analysis of the results.

In Fig. 13a of Nyquist and Bode, the diagram log |Z| vs. log of frequency in Fig. 13b shows impedance data obtained for AISI 316L bare steel. It can be seen that as a function of the exposure time to the corrosive electrolyte, there is an increase in the slope of the curve Zvs. Z in the Nyquist diagram and in the resistance of the solution (data at high frequencies) in Fig. 13b, as well as an order of magnitude in the impedance module, which is related to the formation of the layer of chromium oxide, Cr${ }_{2}$ O${ }_{3}$, which protects the steel from the action of NaCl, increasing its resistance to corrosion. These results are also evident from the increase of the plateau zone by two orders of magnitude of frequency of (10^1^–10^−2^ Hz) after 24 h, as seen in Fig. 13c, while the phase angle remains constant.

For the ZrO${ }_{2}$-ZrO${ }_{x}$N${ }_{y}$-316L system, from the Nyquist diagram (Fig. 14a) it can be seen that after 1 h of immersion in NaCl, there is a decrease in the magnitude of Z, which shows chemical changes in the coating and could be associated with a loss of corrosion resistance. However, the Bode diagram, Fig. 14b and c, shows that the impedance and the phase angle do not undergo significant variations, which indicates little degradation of the coating as a function of immersion time.

The formation of a plateau zone from low to high frequency, Fig. 14c, is indicative of the high capacitive response of the coating and its stability, in comparison with its initial condition. The phase-angle shift at high frequencies as the exposure time increases shows an increase in the corrosion resistance of the coating, showing little degradation of the coating.

For the ZrN-ZrO${ }_{x}$N${ }_{y}$-316L system, the results differ from those of steel and ZrO${ }_{2}$-ZrO${ }_{x}$N${ }_{y}$. The Nyquist diagram in Fig. 15a shows constant degradation as a function of the time of the coating exposed to the electrolyte, with a recovery at 168 h. The radius of the semicircle in the Nyquist diagram undergoes a considerable decrease, which translates into a decrease in the polarization resistance (${R}_{p}$) and therefore the corrosion resistance, this result was also observed in the Bode plot (Fig. 15b). In the Bode plot, the coating degradation process is clearer. In Fig. 15c, the Bode plot shows a single time constant or relaxation time ($\tau$) initially, and after 1 h of exposure two relaxation constants are evident, at 10^3^ and 10^−1^ Hz, which confirms the degradation of the coating. The phase angle shift at low frequencies and the appearance of two $\tau$ could be related to the oxidation of the surface layer of the coating. The characteristics of the corrosion process parameters are also affected by the development of a time constant in the low-frequency range of the EIS spectra, with a displacement of 10^−1^ Hz—10^−2^ Hz. After 1 h of immersion, it exhibits significant electrochemical activity. The increase in Z in the Nyquist diagram after 168 h of immersion could indicate that the electrolyte has penetrated the coating, reaching the substrate, as can be seen in Fig. 16, where it can be seen that this value corresponds to that of steel.

When comparing the impedance results obtained for steel (substrate) and the two types of coating at the different exposure times to the corrosive electrolyte, in Fig. 16a and b, it can be seem that during the first hours of immersion, the corrosion resistance for the ZrO${ }_{2}$-ZrO${ }_{x}$N${ }_{y}$-316L system, with an impedance module around 10^8^ Ω cm^2^, is far superior to steel and to the ZrN-ZrO${ }_{x}$N${ }_{y}$-316L system (10^6^ Ω cm^2^).

Initially, an impedance modulus of the ZrO${ }_{2}$-ZrO${ }_{x}$N${ }_{y}$ coating two orders of magnitude higher can be seen compared to steel and ZrN/ZrO${ }_{x}$N${ }_{y}$, the latter being slightly higher than that of the substrate. These results show that the resistance of the coating to being polarized is 100 times greater for ZrO${ }_{2}$-ZrO${ }_{x}$N${ }_{y}$. On the other hand, the magnitude of the phase angle is of the same order of magnitude for steel and for ZrO${ }_{2}$-ZrO${ }_{x}$N${ }_{y}$, with a phase angle ($\theta$) close to 80, which indicates a capacitive behavior with good dielectric properties, while for ZrN-ZrO${ }_{x}$N${ }_{y}$ it is 20° lower. Furthermore, the formation of a plateau zone from low to high frequency two orders of magnitude lower than AISI 316L and very similar for the two types of coating is indicative of the high capacitive response of the coating.

After one hour of immersion in the corrosive electrolyte, the Nyquist plot shows a drastic fall in the arc of the semicircle Z vs. Z for ZrN to values lower than for steel, Fig. 16b. In the Bode diagram, the impedance module, and therefore the resistance to polarization, falls by two orders of magnitude, equaling that of steel. Regarding the phase angle, there is an order of magnitude shift at higher frequencies, and two time constants appear, showing a second relaxation process related to the penetration of the 3.5 wt. % NaCl solution through the network of pores of the coating, which reaches the substrate. Hence a rapid degradation of the ZrN-ZrO${ }_{x}$N${ }_{y}$ coating is evident after 1 h of immersion in NaCl. The process of degradation of the ZrN-ZrO${ }_{x}$N${ }_{y}$ coatings continues after 24 h of immersion and stabilizes after 72 h, Fig. 16c and -d, while the ZrO${ }_{2}$-ZrO${ }_{x}$N${ }_{y}$ coating does not show representative changes with respect to its initial state during the 164 h of immersion, Fig. 16e and f, demonstrating its high stability and resistance to corrosion by the corrosive electrolyte. The resistance to polarization, the plateau zone, and the resistance of the solution, ${R}_{s}$, remain constant.

**Figure 17:**
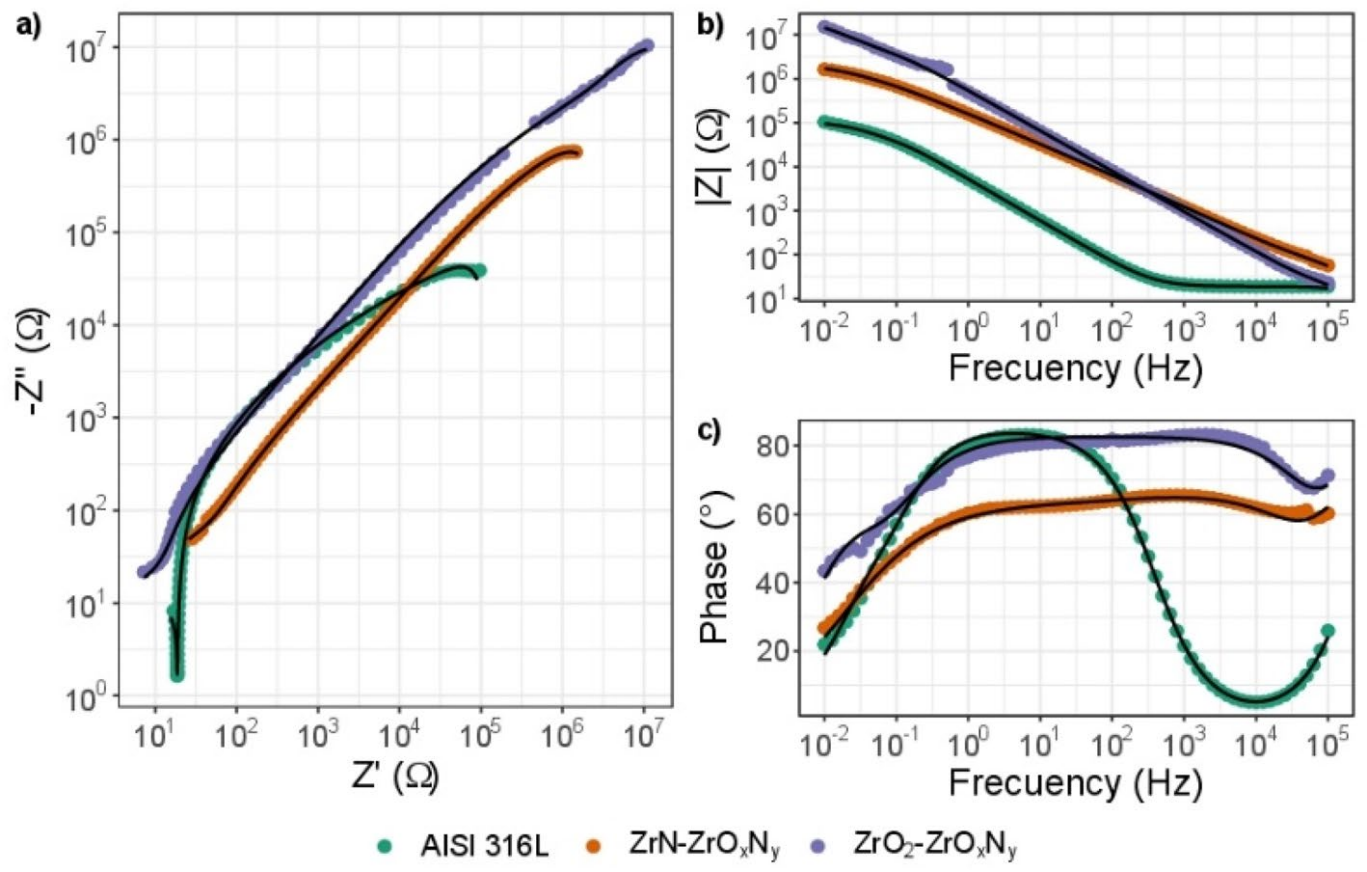
Nyquist diagram obtained from the impedance test for coated and uncoated samples in NaCl solution, (**a**) Nyquist impedance (**b**) Bode and (**c**) Bode plots; the solid line is the fit with the equivalent circuit.

**Figure 18:**
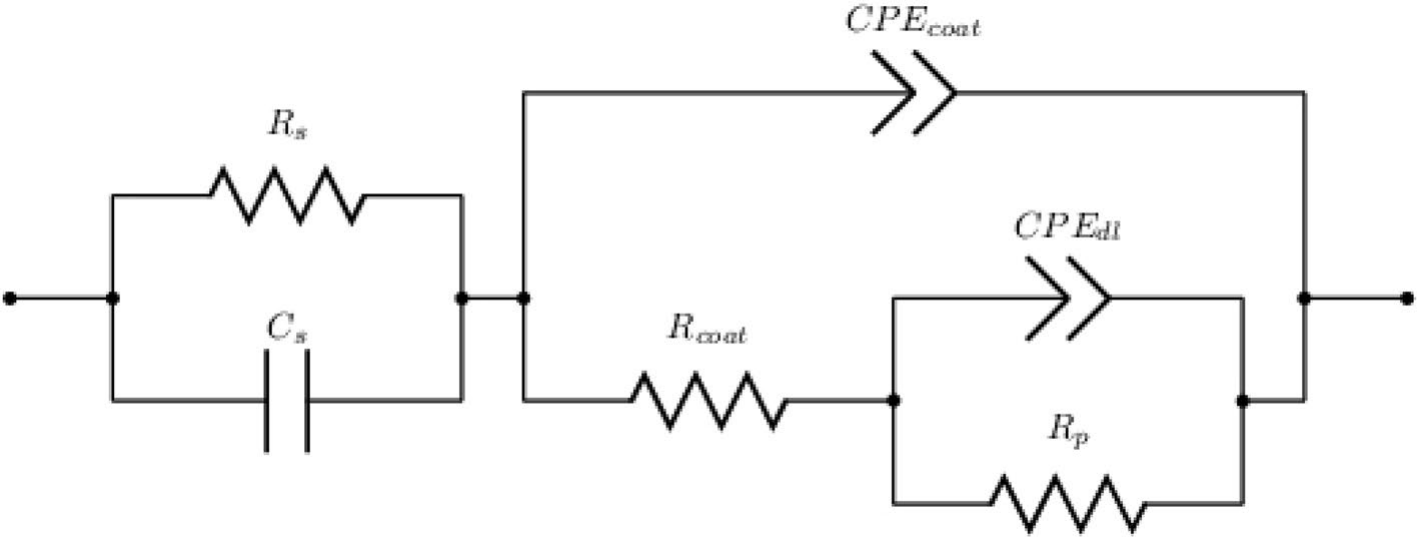
Equivalent electrical circuits used in order to modeling the data measured in the EIS diagram for coated samples.

In Fig. 17, the impedance spectra for the initial immersion in 3.5 wt. % NaCl solution of ZrN-ZrO${ }_{x}$N${ }_{y}$ and ZrO${ }_{2}$-ZrO${ }_{x}$N${ }_{y}$ coatings are compared against AISI 316L. Table 4 shows data extracted from the equivalent electrical circuit in Fig. 18. The chi-square values determined by ZView software are usually on the order of ${10}^{-3}$, which means a good agreement for the curve fitted between the measured and calculated values by using the equivalent circuits. The equivalent electrical circuit used to analyze the impedance spectrum can be shown as a parallel circuit (RC) with resistance R, and capacity C; coupled in series with a parallel circuit for the solution. The surface heterogeneity caused by microcracks, defects in the crystal lattice, roughness, the presence of impurities and porosity is represented by the constant phase element (CPE) (Fig. 18). $CP{E}_{coat}$ and $CP{E}_{dl}$ represent the constant phase element of the coating and the capacitance of double layer, respectively. ${R}_{coat}$ represents the resistance of the coating, ${R}_{p}$ the polarization resistance, and ${R}_{s}$ the solution resistance. The $CPE$ is used instead of the capacitive element to achieve a more accurate fit with the experimental data. In Table 4, the values of the EIS parameters, ${R}_{s}$ and ${R}_{p}$, are reported, which are derived from appropriate equivalent electrical circuits using a constant phase element.

**Table 4:** Electrochemical parameters obtained from equivalent electrical circuits for the coating vs stainless steel.

Sample	${R}_{s}$(Ω)	${C}_{s}$(F)	$CP{E}_{coat}-T$(Ω^−1^ s^n^)	*${n}_{coat}$	${R}_{coat}$(Ω)	$CP{E}_{dl}-T$(Ω^−1^ s^n^)	*${n}_{dl}$	${R}_{p}$(Ω)
AISI 316L	18.81	3.66 × 10^−8^	3.31 × 10^−5^	0.948	6.79 × 10^4^	8.60 × 10^−5^	0.744	4.65 × 10^5^
ZrO_2_-ZrO_*x*_N_*y*_	10.24	1.40 × 10^−7^	3.39 × 10^−7^	0.919	7.45 × 10^6^	5.59 × 10^−7^	0.901	1.68 × 10^7^
ZrN-ZrO_*x*_N_*y*_	29.10	3.47 × 10^−8^	2.52 × 10^−8^	0.975	1.20 × 10^2^	1.72 × 10^−6^	0.683	2.44 × 10^6^

It can be seen in Fig. 17a that the semicircular diameter the Nyquist plot of the ZrO${}_{2}$-ZrO${}_{x}$N${}_{y}$ coatings was larger than that of the ZrN-ZrO${}_{x}$N${}_{y}$ coatings and AISI 316L. According to the Bode graph in Fig. 17b, the values of ${R}_{s}$ (shown in Table 4) for the ZrO${ }_{2}$-ZrO${ }_{x}$N${ }_{y}$, ZrN-ZrO${ }_{x}$ N${ }_{y}$ and AISI 316L, coatings were 10.24, 29.10 and 18.81 Ω, respectively. The polarization resistance (${R}_{p}$) of ZrO${ }_{2}$-ZrO${ }_{x}$N${ }_{y}$ (1.68 × 10^7^) > ZrN-ZrO${ }_{x}$N${ }_{y}$ (2.44 × 10^6^) > AISI 316L (4.65 × 10^4^) showed the higher phase impedance (Z) and greater ${R}_{p}$, respectively, in which the ZrO${ }_{2}$-ZrO${ }_{x}$N${ }_{y}$ coating was thicker and denser. Its high capacity is shown in Fig. 17a. ZrO${ }_{2}$-ZrO${ }_{x}$N${ }_{y}$ film exhibited barrier properties during the EIS test and resulted in a higher polarization resistance in the NaCl solution. Therefore, the corrosion resistance of AISI 316L can be significantly improved by the ZrO${ }_{2}$-ZrO${ }_{x}$N${ }_{y}$ coating.

The results show that charge transfer resistance of the AISI 316L substrate was greatly increased from 6.79 × 10^4^ to 7.45 × 10^6^ Ω when the steel was coated with ZrO${ }_{2}$-ZrO${ }_{x}$N${ }_{y}$, while that of ZrN is lower by two orders of magnitude, which could be related to the micropores and microcracks observed via SEM. Diffusional impedance is primarily related to the microstructure of coatings; a morphology with more porosity and deep grain boundaries can provide efficient diffusion channels that facilitate the passage of the corrosive electrolyte through the coating. In addition, CPEcoat capacity decreased by two orders of magnitude and $n=0.919$, indicating the ideal capacitive behavior of ZrO${ }_{2}$-ZrO${ }_{x}$N${ }_{y}$ (when $n=1$, the CPE behaves like a pure capacitor; if $n=0$, the behavior is that of a resistor). This behavior is increased by that of the double layer, where ZrO${ }_{2}$-ZrO${ }_{x}$N${ }_{y}$ has lower resistance to charge transfer and the size of n is closer to 1 ($n=0.902$).

### Biocompatibility study

The efficiency of a coating to be used in a bone implant is associated with the morphology of the surface and the physical–chemical interaction of the bone biomaterial; these are essential characteristics for good osseointegration. The two types of coating evaluated here, ZrO${ }_{2}$-ZrO ${ }_{x}$N${ }_{y}$ and ZrN-ZrO${ }_{x}$N${ }_{y}$, meet these requirements and are nanoceramics deposited on surgical-grade stainless steel. However, the morphology of the ZrN-ZrO${ }_{x}$N${ }_{y}$ coating deposited on AISI 316L post-corrosion showed delamination of the coating during the corrosion test, which could lead to allergic reactions generated by its release into the bloodstream. Therefore, only the ZrO${ }_{2}$-ZrO${ }_{x}$N${ }_{y}$-316L coating was used for biocompatibility tests, using the MTT method and direct counting of cells using confocal fluorescence microscopy.

**Figure 19:**
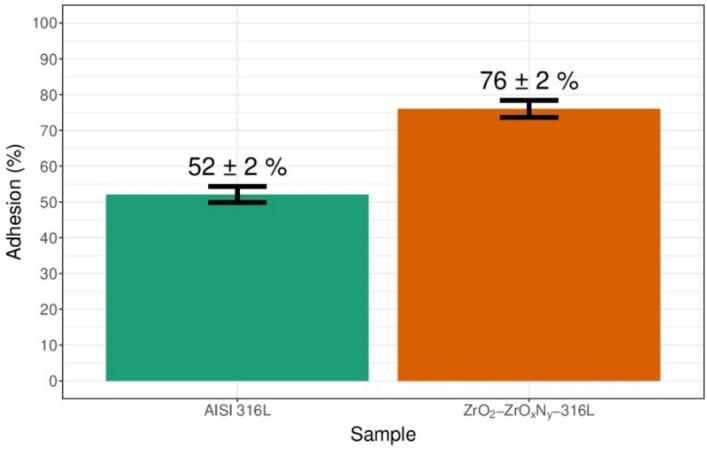
Percentage of cell adhesion of osteoblast on AISI 316L and ZrO_2_-ZrO_*x*_N_*y*_-316L system. DAPI-stained cells on coated and uncoated stainless steel were counted using fluorescence and percentage was determined with the number of adhered cells divided by total cell number seeded in each well.

MTT is a yellow aqueous solution, which, on reduction by dehydrogenases and reducing agents present in metabolically active cells, yields water-insoluble violet-blue formazan crystals that can be extracted with organic solvents and quantified by spectrophotometry. The amount of formazan is directly proportional to the number of living cells, and because of this, the MTT method is widely used to assess cytotoxicity and cell viability. Figure 19 shows the results obtained by means of the MTT technique, from the culture of bone cells on 316L stainless steel and on the same steel coated with ZrO${ }_{2}$-ZrO${ }_{x}$N${ }_{y}$, finding 27% higher growth for cells grown on the ZrO${ }_{2}$-ZrO${ }_{x}$N${ }_{y}$ coating than on bare steel.

**Figure 20:**
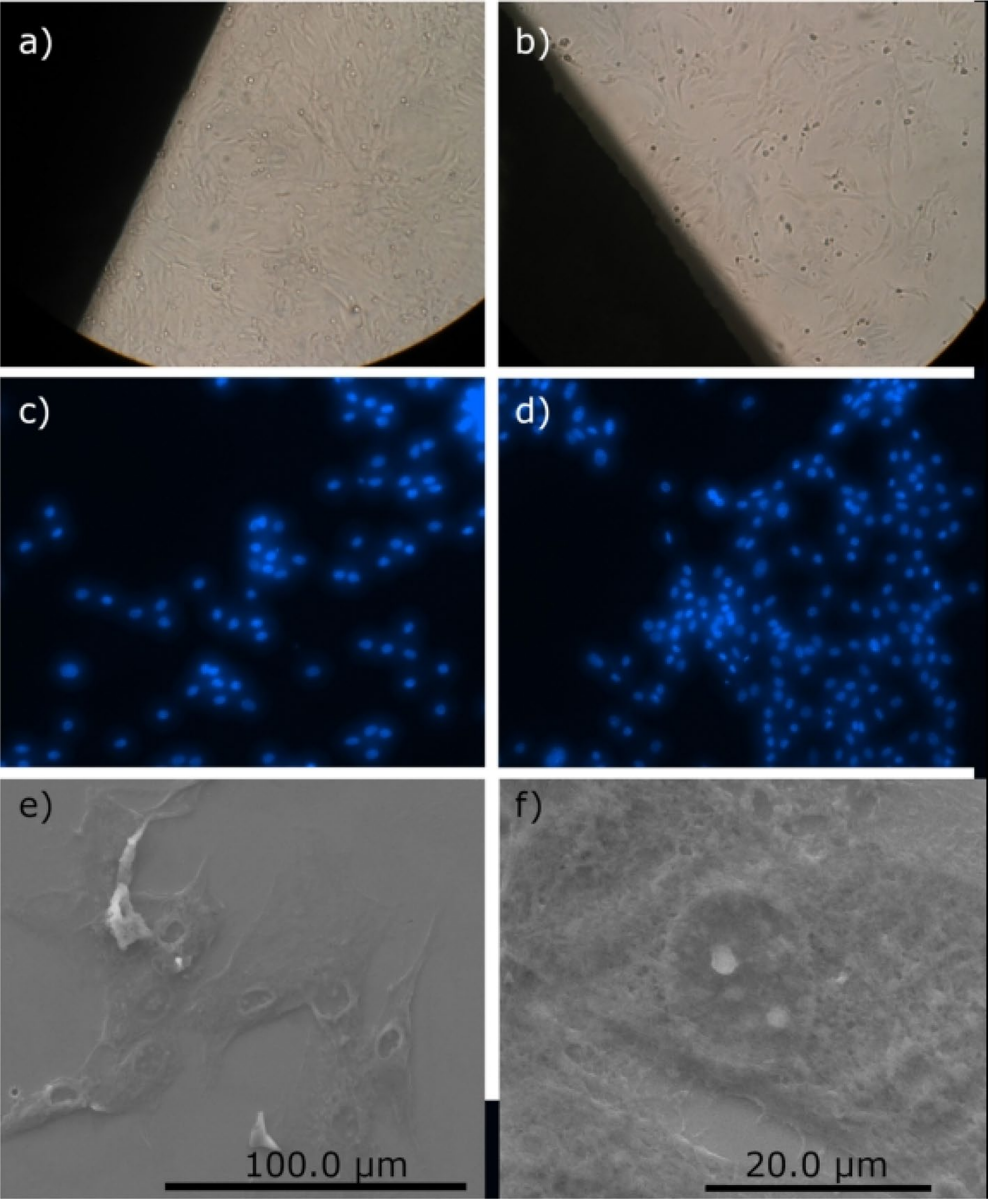
Optical micrograph of the proliferation of osteoblast primary (**a**) uncoated AISI 316L and (**b**) ZrO_2_-ZrO_*x*_N_*y*_-316L system at 72 h. Images of fluorescence microscopy (**c**) uncoated AISI 316L and (**d**) ZrO_2_-ZrO_*x*_N_*y*_-316L system at 72 h with a filter; (**e**) and (**f**) culture cells derived from the mouse cranial vault C57BL/6 at 24 h on ZrO_2_-ZrO_*x*_N_*y*_-316L system.

Optical micrographs of primary osteoblast cells on bare steel and on the ZrO${ }_{2}$-ZrO${ }_{x}$N${ }_{y}$-316L system can be seen in Fig. 20a and b, respectively. Cell proliferation on coated and uncoated stainless steel was followed by nuclei staining and observation with fluorescence microscopy of mouse bone cells grown for 72 h on the samples of untreated 316 L steel (Fig. 20c) and 316 L steel coated with ZrO${ }_{2}$-ZrO${ }_{x}$N${ }_{y}$ film (Fig. 20d). Live cell count per field indicated bone cell growth greater than 64% on coated steel compared to bare steel. Additionally, through scanning electron microscopy on cells cultured for 24 h to avoid cell confluence, the cell morphology exhibited a characteristic growth of spindle cells, elongated and thin (Fig. 20e and f), with good adherence to the substrate, which indicates that they are active bone cells and that the layer of ZrO${ }_{2}$-ZrO${ }_{x}$N${ }_{y}$ has the ability to induce the attachment, propagation, proliferation, and growth of osteoblasts on its surface. Similar results have been reported for thin TiN films deposited on NiTi alloy and for zirconia-based nanoceramics and TiCuN solid-solution coating^20,45,46^.

Nanoceramic coatings are known to promote osteochondral formation followed by osteogenesis and vascularization, and the development of bone requires several sequences of processes, such as the adhesion of osteogenic cells followed by their survival and multiplication. The studied ZrO${ }_{2}$-ZrO${ }_{x}$N${ }_{y}$ coating, with a roughness of 9.6 nm and a particle size of 381 nm (Table 2), meets these requirements, and therefore, it would contribute to the improvement of the proliferation and adherence of the osteoblasts, which will lead to better biocompatibility compared to uncoated AISI 316L, giving valuable information on the potential use of the coating in osteosynthesis processes. The ZrO${ }_{2}$-ZrO${ }_{x}$N${ }_{y}$-316L system guarantees resistance to the traction forces generated by the cells during their adhesion process, a barrier that cannot be overcome by highly soft and deformable substrates, where cells cannot adhere, spread, and survive. Additionally, its good corrosion resistance is indicative of the protective character of the coating in media rich in chlorides. D. Roman et al.^42^ also evaluated the corrosion resistance of a zirconium nitride coating deposited onto titanium and concluded that the protective effect exerted by the coating on titanium is due to the formation of a ZrO${ }_{2}$-ZrO${ }_{x}$N${ }_{y}$ amorphous mixture.

On the other hand, when a biocompatible coating is studied, not only must its ability to promote cell proliferation be evaluated, but also its resistance to corrosion in a corrosive medium such as blood plasma, where the presence of chlorides favors pitting corrosion. The electrolytes in contact with the coating in vivo generate pitting corrosion, and it is precisely these corrosion products that can trigger the immune system and trigger allergic reactions in vivo. For this reason, the morphology of the coating, its chemical composition, and its resistance to corrosion are important properties for the evaluation of a biocompatible coating^45,47^.

The difference in the results for the cell proliferation obtained via MTT and confocal fluorescence microscopy can be explained by the fact that MTT tetrazolium reduction primarily depends on the rate of glycolytic NADH production in the endoplasmic reticulum (ER), where collateral reactions may occur, while microscopy allows directly quantifying living cells.

## Conclusions

Using unbalanced DC magnetron sputtering, coatings of ZrN-ZrO${ }_{x}$N${ }_{y}$ and ZrO${ }_{2}$-ZrO${ }_{x}$N${ }_{y}$-316L with cubic crystalline structures and preferential in-plane growth (222) and (111), respectively, were deposited onto stainless steel. The depth profile of the composition of the coating indicates that it is oxidized to zirconia on the surface, but its composition from the surface to the substrate is homogeneous and consists of a mixture of ZrN-ZrO${ }_{x}$N${ }_{y}$ phases for coatings deposited in an N${ }_{2}$/O${ }_{2}$ 95/05 atmosphere and ZrO${ }_{2}$-ZrO${ }_{x}$N${ }_{y}$ phases for coatings deposited in an N${ }_{2}$/O${ }_{2}$ 79/21 atmosphere.

The corrosion resistance of the coated steel is greater than that of bare steel in the following order: ZrO${ }_{2}$-ZrO${ }_{x}$N${ }_{y}$ > ZrN-ZrO${ }_{x}$N${ }_{y}$ > stainless steel, where ZrN-ZrO${ }_{x}$N${ }_{y}$-316L exhibits great damage to its post-corrosion surface morphology compared to ZrO${ }_{2}$-ZrO${ }_{x}$N${ }_{y}$-316L. These results were confirmed via EIS.

The biocompatibility study, evaluated by the growth of mouse bone cells, shown as live cell count per field, indicated growth 64% greater on ZrO${ }_{2}$-ZrO${ }_{x}$N${ }_{y}$-316L than on bare steel. These results indicate that the ZrO${ }_{2}$-ZrN${ }_{x}$O${ }_{y}$ coating promotes the adhesion of mouse bone cells, facilitating their proliferation.
